# Tomato nuclear proteome reveals the involvement of specific E2 ubiquitin-conjugating enzymes in fruit ripening

**DOI:** 10.1186/s13059-014-0548-2

**Published:** 2014-12-03

**Authors:** Yuying Wang, Weihao Wang, Jianghua Cai, Yanrui Zhang, Guozheng Qin, Shiping Tian

**Affiliations:** Key Laboratory of Plant Resources, Institute of Botany, Chinese Academy of Sciences, No.20 Nanxincun, Xiangshan, Haidian District, Beijing, 100093 China; The Graduate University of the Chinese Academy of Sciences, Yuquanlu, Beijing, 100049 China

## Abstract

**Background:**

Fruits are unique to flowering plants and play a central role in seed maturation and dispersal. Molecular dissection of fruit ripening has received considerable interest because of the biological and dietary significance of fruit. To better understand the regulatory mechanisms underlying fruit ripening, we report here the first comprehensive analysis of the nuclear proteome in tomato fruits.

**Results:**

Nuclear proteins were isolated from tomatoes in different stages of ripening, and subjected to iTRAQ (isobaric tags for relative and absolute quantification) analysis. We show that the proteins whose abundances change during ripening stages are involved in various cellular processes. We additionally evaluate changes in the nuclear proteome in the ripening-deficient mutant, *ripening-inhibitor* (*rin*), carrying a mutation in the transcription factor RIN. A set of proteins were identified and particular attention was paid to SlUBC32 and PSMD2, the components of ubiquitin-proteasome pathway. Through chromatin immunoprecipitation and gel mobility shift assays, we provide evidence that RIN directly binds to the promoters of *SlUBC32* and *PSMD2*. Moreover, loss of RIN function affects protein ubiquitination in nuclei. *SlUBC32* encodes an E2 ubiquitin-conjugating enzyme and a genome-wide survey of the E2 gene family in tomatoes identified five more E2s as direct targets of RIN. Virus-induced gene silencing assays show that two E2s are involved in the regulation of fruit ripening.

**Conclusions:**

Our results uncover a novel function of protein ubiquitination, identifying specific E2s as regulators of fruit ripening. These findings contribute to the unraveling of the gene regulatory networks that control fruit ripening.

**Electronic supplementary material:**

The online version of this article (doi:10.1186/s13059-014-0548-2) contains supplementary material, which is available to authorized users.

## Background

Fruits represent important components of human diets, providing essential vitamins and a wide range of ‘bioactive’ compounds important for human health, such as carotenoids, polyphenols, plant sterols, and polyunsaturated fatty acids [1]. The ripening of fruits, a genetically programmed process, has received considerable attention because of the specificity of this developmental process to plant biology and the important impact of ripening on fruit quality and shelf life. Fruit ripening is regulated by both internal and external cues, including hormones, developmental genes, light, and temperature [2]. Due to the different ripening mechanisms, fruits are classically divided into two groups; climacteric, which are characterized by an increase in respiration and a concomitant burst of phytohormone ethylene at the onset of ripening, and non-climacteric, which do not exhibit increased respiration and typically produce little ethylene during ripening.

Ethylene plays crucial role on ripening of climacteric fruits [3-6], and great strides have been made toward ethylene biosynthesis and ethylene signal transduction pathways [7-9]. However, ripening of non-climacteric fruit is thought to be ethylene independent. The discovery of genes underlying rare spontaneous mutations in tomatoes that completely abolish the normal ripening process has revealed primary ripening control upstream of ethylene. These mutations include the *ripening-inhibitor* (*rin*), *non-ripening* (*nor*), and *Colorless non-ripening* (*Cnr*). All the *rin*, *nor*, and *Cnr* loci harbor transcription factor genes. The *rin* locus encodes a MADS-box transcription factor termed MADS-RIN [10], while *nor* encodes a member of the NAC-domain transcription factor family [11]. The *Cnr* locus encodes a SQAMOSA promoter binding (SPB) protein [12]. These proteins might represent conserved genetic regulators that are shared among climacteric and non-climacteric fruits. Additional tomato transcription factor genes, including *TOMATO AGAMOUS-LIKE1* (*TAGL1*), *HD-ZIP HOMEOBOX PROTEIN-1* (*HB-1*), *APETALA2a* (*AP2a*), *ETHYLENE RESPONSE FACTOR6* (*ERF6*), *ARABIDOPSIS PSEUDO RESPONSE REGULATOR2-LIKE* (*APRR2-Like*), and two *FRUITFULL* homologs (*TDR4/FUL1* and *MBP7/FUL2*), have been demonstrated to play vital roles in fruit ripening [13-22]. Recently, it was proposed that DNA methylation contributes to the regulation of fruit ripening [12,23], and the methylomes of tomato fruits from immature to fully ripe were profiled [23]. The regulatory process of fruit ripening by either transcription factor or DNA methylation occurs in nucleus, implying the involvement of novel potential nuclear proteins in fruit ripening.

The nucleus is the most prominent organelle that contains majority of the genetic materials, and is essential for gene expression and regulation. About 10% to 20% of the total cellular proteins are predicted to be localized in the eukaryotic nucleus. Nuclear proteins constitute a highly organized but complex network that performs diverse functions during development and physiological processes. In recent years, high-throughput nuclear proteome analysis has been performed in various plants, including *Arabidopsis* [24], rice [25], chickpea [26], and *Medicago* [27], to analyze the function of specific nuclear proteins. Organelle proteomics is a promising strategy that reduces the complexity of the total cellular proteome, focusing on a specific group of proteins that are central to the biological process under investigation [28-30]. At present, however, knowledge regarding the global expression profile of nuclear proteins during fruit ripening is still lacking. Although the transcript levels of putative nuclear genes were revealed by several high-throughput transcriptome studies [31,32], it is insufficient to predict the corresponding protein abundances, since the level of mRNA does not always correlate well with the level of protein [33-35]. Expression levels of a protein are decided not only by transcription rates of the gene, but also by other control mechanisms, such as nuclear export and mRNA localization, transcript stability, translational regulation and protein degradation [36]. Furthermore, the activity and the function of proteins can be altered through post-translational modifications (for example, phosphorylation and glycosylation) or targeted proteolysis [36]. Therefore, proteome studies could complement the transcriptome analyses. Characterization of the nuclear proteome in fruit ripening holds the promise to understand the molecular basis of the ripening process.

In the present study, we performed a quantitative analysis of nuclear proteome during tomato fruit ripening. Nuclear proteins were isolated from tomato fruits in four stages of ripening, from mature green to red ripe, and analyzed by the advanced isobaric tags for relative and absolute quantification (iTRAQ) technology coupled with NanoLC-MS/MS. Furthermore, we evaluated changes in the nuclear proteome in the *rin* mutant. A number of proteins were identified and particular attention was paid to proteins involved in ubiquitin-proteasome pathway. Further analysis indicated that RIN directly regulated the expression of several genes encoding ubiquitin E2 enzymes during tomato fruit ripening. Specific E2 genes were demonstrated to be involved in the regulation of fruit ripening based on virus-induced gene silencing (VIGS) assays.

## Result

### Tomato nuclei enrichment and purity assessment

**Figure 1:**
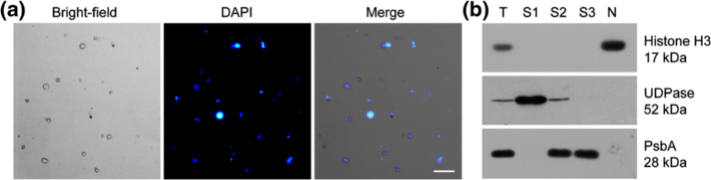
**Preparation of tomato nuclei for proteomic analysis. (a)** Micrographs showing representative nuclear fractions from tomato fruits after 4′,6-diamidino-2-phenylindole (DAPI) staining. The phase-contrast micrograph and the fluorescence micrograph of the nuclei are presented. Scale bar, 25 μm. **(b)** Western blot analysis of the different purification fractions with antibodies directed against histone H3, UDP-glucose pyrophosphorylase (UGPase), and photosystem II reaction centre protein D1 (PsbA). T, total protein extract; S1, supernatant fraction from centrifugation at 3,000 × *g*; S2, supernatant fraction after 1% Triton X-100 treatment and centrifugation; S3, supernatant fraction from sucrose density centrifugation; N, nuclear protein extract.

Purification of nuclei away from other cellular contaminants is vital to nuclear subproteome analysis. We isolated intact nuclei from tomato fruit using differential centrifugation and sucrose density enrichment. The integrity of the isolated nuclei was assessed using 4′,6-diamidino-2-phenylindole (DAPI) staining and examined by fluorescence microscopy (Figure 1a). The nuclei were uniform spheres with an average diameter of approximately 10 μm. To further evaluate the enrichment and purity of the nuclei, western blotting was performed with antibodies against organelle specific proteins. The nuclear protein histone H3 was detected in the nuclear fraction, but not in the cytoplasmic fraction. By comparison, the cytoplasmic protein UDP-glucose pyrophosphorylase (UDPase) and the chloroplast protein photosystem II reaction center protein D1 (PsbA), which are absent from the nuclei, were not found in the purified nuclear fraction (Figure 1b). These data suggest that the nuclei were successfully enriched and there was no appreciable level of contamination by chloroplast or cytoplasm in the isolated nuclei. Nuclear proteins were prepared from the nuclei-enriched fraction using a phenol-based method to avoid contamination by nucleic acids.

### Quantitative proteomic analysis reveals the changes in abundance of nuclear proteins during fruit ripening

**Figure 2:**
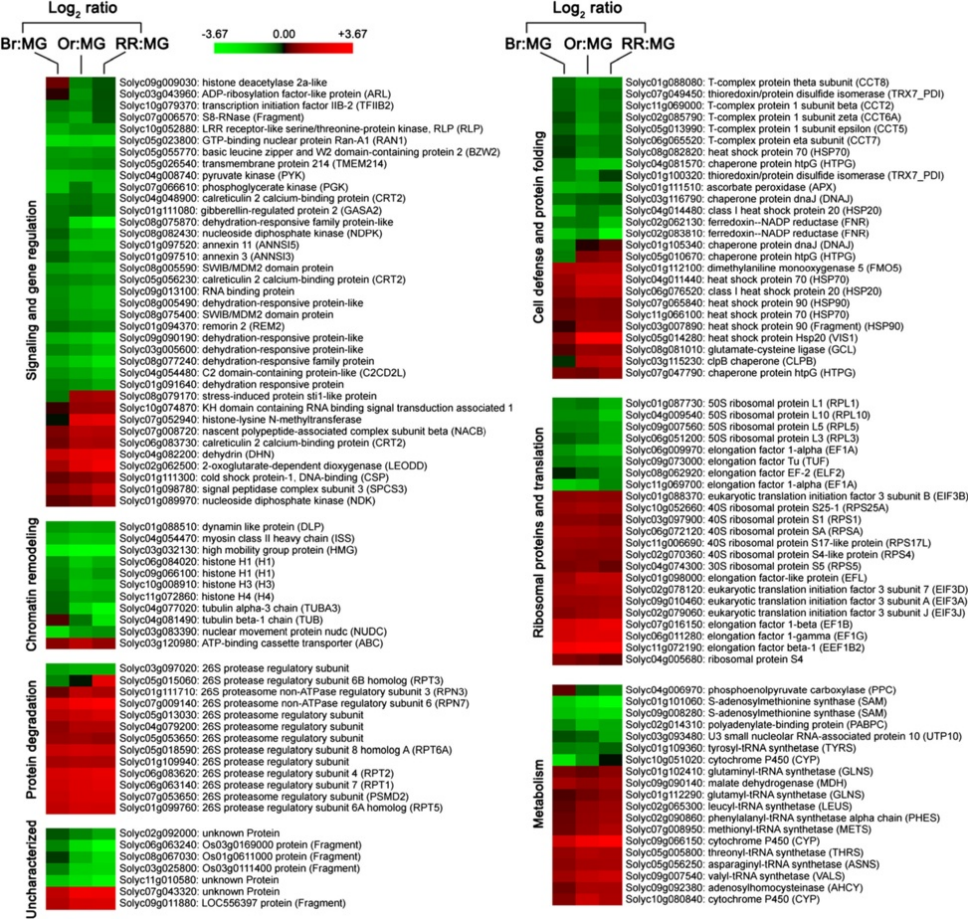
**Quantitative analysis of nuclear proteome during tomato fruit ripening.** Nuclear proteins were extracted from tomato fruits at mature green (MG), breaker (Br), orange (Or), and red ripe (RR) ripening stages, and subjected to isobaric tags for relative and absolute quantification (iTRAQ) labeling coupled with NanoLC-MS/MS. The 136 differentially expressed proteins were classified into seven functional categories and the expression patterns within each were hierarchically clustered. Expression ratios were calculated using the earlier ripening stage, that is, MG, as denominator, and plotted in a heat map on a log_2_ scale. Each row in the color heat map indicates a single protein, and the gene identifiers (Solyc numbers) and functional annotations are shown. The green and red colors indicate down- and upregulation, respectively, in an indicated ripening stage relative to the MG stage. Black represents no significant expression change. Data from biologically repeated samples are averaged and the detailed information on proteins is listed in Additional file 2.

An iTRAQ-based quantitative proteomic analysis was utilized to gain a global view of nuclear proteome alteration during tomato fruit ripening. An overview of the iTRAQ experimental design and the workflow is depicted in Additional file 1. The nuclear proteins were isolated from tomatoes in four stages of ripening, that is, mature green, breaker, orange, and red ripe stages. Simultaneous comparison of nuclear protein expression across these samples was achieved using four-plex iTRAQ isobaric tags with NanoLC-MS/MS. Two independent biological replicates for each sample were used for labeling. Using the *Solanum lycopersicum* protein database, a total of 1,279 and 1,303 proteins were identified with a global false discovery rate (FDR) below 1% in the two biological replicates, respectively. These identified proteins were filtered to calculate the meaningful cutoff using a population statistics applied to the biological replicates as proposed by Gan *et al.* [37]. The cutoff values were then used to verify whether the changes in protein abundance are significant. A total of 136 proteins were finally screened as significantly altered at one or more ripening stages. Additional file 2 shows these differentially expressed proteins along with all relevant identification information and the ratio of iTRAQ reporter ion intensities. According to the Functional Catalogue (FunCat) annotation scheme [38] and the UniProt Knowledgebase (UniProtKB) [39], these proteins were classified into seven functional categories, namely signaling and gene regulation, chromatin remodeling, protein degradation, cell defense and protein folding, ribosomal proteins and translation, metabolism, and uncharacterized. To identify the proteins showing similar expression profiles, hierarchical clustering [40] was applied within each functional category (Figure 2).

The largest functional class, ‘signaling and gene regulation’, was associated with cell signaling and transcription regulation. Thirty-seven proteins with differential regulation were identified in this category, including GTP-binding nuclear protein Ran-A1 (Solyc05g023800), basic leucine zipper and W2 domain-containing protein 2 (Solyc05g055770), and transcription initiation factor IIB-2 (Solyc10g079370). Notably, histone-lysine N-methyltransferase (Solyc07g052940) and histone deacetylase 2a-like (Solyc09g009030), which participate in histone modifications, were also identified in this functional category. Histone-lysine N-methyltransferase was upregulated during fruit ripening, whereas histone deacetylase 2a-like was downregulated. Histone modifications play an important role in transcription regulation [41], but their functions in fruit ripening remain largely unknown. In addition, we successfully identified 11 proteins involved in chromatin remodeling, such as nuclear movement protein nudc (Solyc03g083390), high mobility group protein (Solyc03g032130), and histone H1 (Solyc06g084020 and Solyc09g066100), H3 (Solyc10g008910) and H4 (Solyc11g072860). All of the identified histones were downregulated during fruit ripening. The ‘cell defense and protein folding’ class was composed largely of molecular chaperones, which are well known for their roles in preventing protein aggregation and for regulating the activity of many signal transduction proteins. The expression of these proteins exhibited diverse patterns. Furthermore, we identified proteins involved in protein degradation, translation, and metabolism. These proteins were differentially regulated during fruit ripening.

### Expression of nuclear proteins are altered in the *rin* mutant

**Figure 3:**
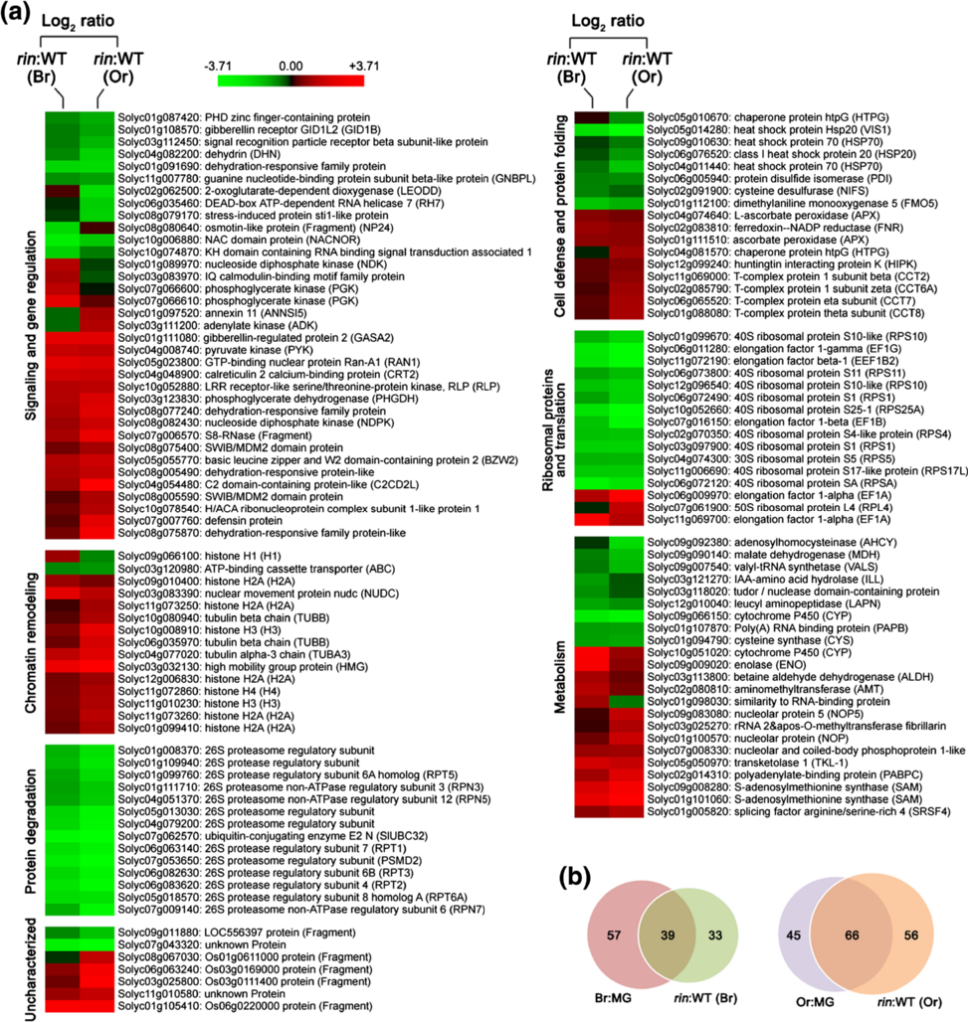
**Changes in nuclear proteome in** ***rin*** **mutant reveal the potential downstream targets of RIN. (a)** Nuclear proteins were isolated from wild-type (WT) and *rin* mutant fruits at breaker (Br) and orange (Or) ripening stages, and subjected to isobaric tags for relative and absolute quantification (iTRAQ) labeling coupled with NanoLC-MS/MS. A total of 127 proteins showing differential expression in the *rin* mutant relative to the wild-type at Br or Or stage were identified and classified into seven functional categories. The expression patterns of the proteins within each functional category were hierarchically clustered based on the expression ratio as a log_2_ scale. Each row in the color heat map indicates a single protein. The gene identifiers (Solyc numbers) and the functional annotations are shown. The green and red colors indicate down- and upregulation, respectively, in the *rin* mutant relative to the wild-type. Black represents no significant expression change. Data from biologically repeated samples are averaged and the detailed information on proteins is listed in Additional file 4. **(b)** Venn diagram showing the overlap of proteins that changed abundance in the process of fruit ripening and those that changed abundance in the *rin* mutant at the same ripening stage (Br or Or).

Transcription factor RIN represents a global developmental regulator of fruit ripening. To further dissect the complex networks of ripening-related pathways, nuclear protein extracts from wild-type and *rin* mutant at breaker as well as orange ripening stages were analyzed in iTRAQ experiments with two independent biological replicates (Additional file 3). In total, 1,379 and 1,339 proteins were identified in the two biological replicates, respectively. The meaningful cutoff was calculated following the method of Gan *et al.* [37] to assess whether the changes in protein abundance are significant. A total of 127 proteins were found to experience significant up- or downregulation in the *rin* mutant at one ripening stage or both (Additional file 4). These proteins were classified into seven functional categories as described above, and the differential expression patterns within each were hierarchically clustered (Figure 3a).

The functional class ‘signaling and gene regulation’ represents the largest category and proteins in this class were differentially regulated in the *rin* mutant. Several transcription factors, namely SWIB/MDM2 domain protein (Solyc08g005590 and Solyc08g075400) and NAC domain protein (Solyc10g006880), were identified in this class. While SWIB/MDM2 domain protein was upregulated in the *rin* mutant, NAC domain protein was downregulated. Transcription factors play crucial roles in fruit ripening [10-12]. However, due to low copy numbers, transcription factors are difficult to be detected by mass spectrometry. The function of these transcription factors identified in our study deserves further research. Strikingly, we identified 14 proteins in the ‘protein degradation’ class, whose expression was consistently downregulated in the *rin* mutant. Thirteen of these proteins are members of 26S proteasome regulatory subunits, and one belongs to the ubiquitin-conjugating enzyme E2. All of these proteins are involved in the same molecular pathway, the ubiquitin-proteasome system, which is responsible for removing most abnormal peptides and short-lived cellular regulators. The ubiquitin-proteasome system has been shown to regulate many important biological processes, but little is known about its function in fruit ripening.

When comparing proteins which changed abundance in the process of fruit ripening (Figure 2) alone with those which changed abundance in the *rin* mutant (Figure 3a) at the same ripening stage (breaker or orange), we found a considerable overlap in protein identifiers between them (Figure 3b). This is to be expected as many proteins affected by ripening process will also be affected by the *rin* mutation which effectively blocks most ripening phenomena.

### Genes involved in ubiquitin-proteasome system are identified as direct targets of RIN

**Figure 4:**
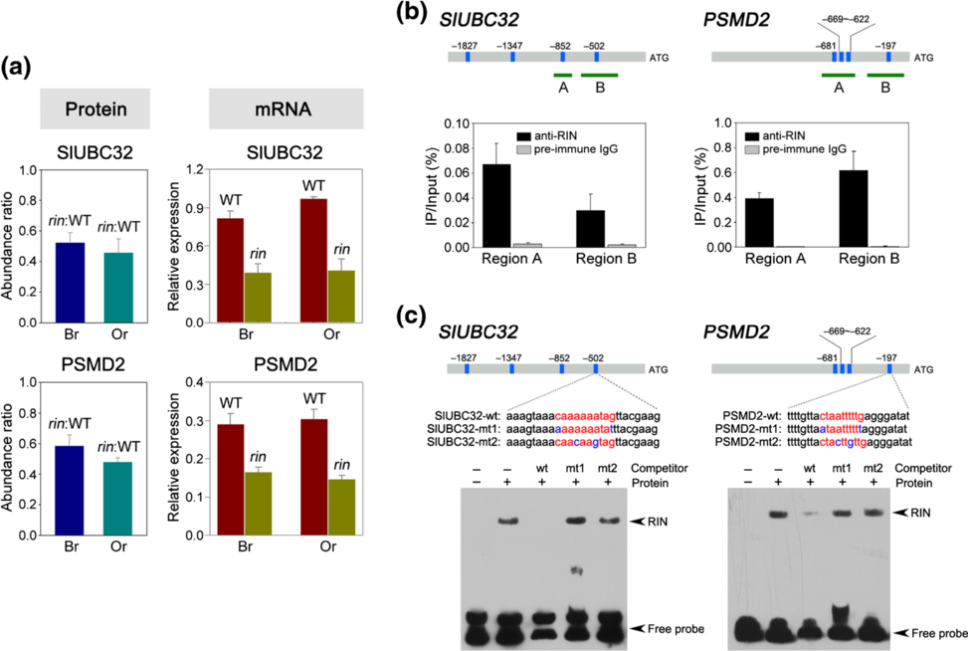
**Genes involved in ubiquitin-proteasome pathway are identified as direct RIN targets. (a)** Expression analysis of SlUBC32 and PSMD2 at protein and mRNA levels. The protein expression in wild-type (WT) and *rin* mutant was assessed by quantitative proteome analysis at breaker (Br) and orange (Or) ripening stages. The mRNA expression was examined by quantitative RT-PCR. The gene transcript levels are normalized against the *actin* gene. Values are means ± SD of three independent experiments. **(b)** ChIP-qPCR assays show that RIN direct binds to the promoter regions of *SlUBC32* and *PSMD2*. The promoter structures of the target genes are presented. Blue boxes represent CArG box elements and numbers indicate the position of these motifs relative to the translational start site. Green fragments with upper-case letters represent the regions used for ChIP-qPCR. Values are the percentage of DNA fragments that co-immunoprecipitated with anti-RIN antibodies (black bars) or non-specific antibodies (preimmune rabbit IgG; grey bars) relative to the input DNAs. Error bars represent the SD of three independent experiments. **(c)** Gel mobility shift assays reveal the direct binding of RIN to CArG box elements in the promoter regions of *SlUBC32* and *PSMD2*. The probe sequences corresponding to the *SlUBC32* and *PSMD2* promoters are shown, with red letters representing the CArG box. The mutated bases in the probes are represented by blue letters. wt, probe with intact CArG box element; mt, probe with mutated CArG box element. As competitors, 1,000-fold excess amounts of unlabeled probes were added to the binding reaction. The retarded bands and the free probes are indicated by arrowheads.

In the quantitative analysis of nuclear proteome between wild-type and *rin* mutant, it is noticeable that 14 proteins showing similar expression patterns were identified as proteins involved in the ubiquitin-proteasome system. Two of these proteins, ubiquitin-conjugating enzyme E2 N (SlUBC32; Solyc07g062570) and 26S proteasome regulatory subunit (PSMD2; Solyc07g053650), were subjected to further characterization. SlUBC32 was the only E2 enzyme identified in the iTRAQ analysis, and PSMD2 represented the protein showing the highest changes in protein abundance (0.58 at breaker stage and 0.48 at orange stage) among the 26S proteasome regulatory subunits identified. SlUBC32 and PSMD2 were downregulated in the *rin* mutant at both breaker and orange stages. To examine whether the protein expression patterns were also present at the transcript level, quantitative reverse transcription polymerase chain reaction (RT-PCR) was performed. The results showed that the transcript alterations in the *rin* mutant were in agreement with the protein expression variations for both genes (Figure 4a).

As a transcription factor, RIN could regulate gene expression either directly or indirectly. Thus, we performed a chromatin immunoprecipitation (ChIP) assay to investigate whether RIN regulates the expression of *SlUBC32* and *PSMD2* by directly binding to their promoters *in vivo*. A search for CArG box element (C(C/T)(A/T)(A/T)(A/T)(A/T)(A/T)(A/T)(A/G)G), the DNA binding sites for RIN [42], in the 2,000 bp upstream region starting from the translational start site (ATG) using the PLACE Web Signal Scan [43] revealed four CArG boxes in the promoters of both *SlUBC32* and *PSMD2* (Additional file 5). For the ChIP assay, cross-linked DNA-protein complexes were immunoprecipitated using affinity-purified anti-RIN polyclonal antibody. Specific primers were designed for *SlUBC32* and *PSMD2* to amplify promoter sequences surrounding CArG box binding sites from the immunoprecipitated DNA (Additional file 6). The binding of RIN to promoter fragments was determined as the relative amount of immunoprecipitated DNA fragments versus input DNA fragments. As a positive control, the binding of RIN protein to promoter of *ACC synthase 2*, a known RIN-target gene [42], was performed. Our results indicated that RIN binds to the promoters of *SlUBC32* and *PSMD2 in vivo* (Figure 4b). Further analysis using electrophoretic mobility shift assay (EMSA) with purified recombinant RIN protein confirmed the results of ChIP assay, showing the binding ability of RIN to the promoters of *SlUBC32* and *PSMD2* (Figure 4c). These results suggest a direct regulation of genes involved in the ubiquitin-proteasome pathway by RIN.

### Protein ubiquitination is affected in the *rin* mutant

Our results demonstrated that genes (*SlUBC32* and *PSMD2*) involved in ubiquitin-proteasome system are directly regulated by RIN. We then investigated the changes in protein ubiquitination in the *rin* mutant. Nuclear proteins were prepared from wild-type and *rin* mutant tomatoes at orange ripening stage and the ubiquitinated proteins was immunoprecipitated with anti-Ub P4D1 (Santa Cruz [44]) that recognizes mono- and polyubiquitinated proteins [45]. We used a state-of-the-art technique in quantitative proteomics termed Single Window Acquisition of all Theoretical spectra Mass-Spectrometry (SWATH-MS) [46] to quantify changes in ubiquitinated proteins in the *rin* mutant. SWATH-MS has been successfully applied to measure quantitative changes of *N*-linked glycoproteins [47] and protein interacts [48].

Application of this method resulted in identification of 177 proteins after ubiquitination-based enrichment. Quantitative analysis revealed that 84 of these ubiquitinated proteins changed abundance significantly (*P* <0.05) in the *rin* mutant (Additional file 7). Among these 84 proteins, 51 (60.1%) did not exhibit significant variations in protein expression levels in the mutant revealed by our iTRAQ analysis. In addition, two proteins, polyadenylate-binding protein (Solyc02g014310) and arginine/serine-rich splicing factor (Solyc06g009060), changed in the opposite tendency in SWATH-MS and iTRAQ analyses (Additional file 7). These proteins are proposed to undergo alterations of ubiquitination levels in the *rin* mutant. The proteins experienced downregulation of ubiquitination levels in the mutant include several ribosomal subunits, eukaryotic translation initiation factors, and the heat-shock proteins, which have previously been shown to be ubiquitinated by MS/MS analysis or direct biochemical assays [49,50]. This list also includes two proteins in the ubiquitin-proteasome system, the 26S protease regulatory subunit 6B homolog (Solyc10g084050) and the ubiquitin protein itself (Solyc07g064130), and proteins involved in other biological processes. The proteins whose ubiquitination levels were upregulated in the *rin* mutant include those involved in signal transduction, protein degradation, cell defense, and metabolism (Additional file 7). Particularly notable, we found the ubiquitination level of histone H2B (Solyc11g007920) was increased in the *rin* mutant. Ubiquitination of histone H2A and H2B is known to play a crucial role in chromatin silencing [51]. Taken together, our results suggest that RIN regulates protein ubiquitination in the nuclei.

### RIN alters the expression of a set of E2 genes during fruit ripening

**Figure 5:**
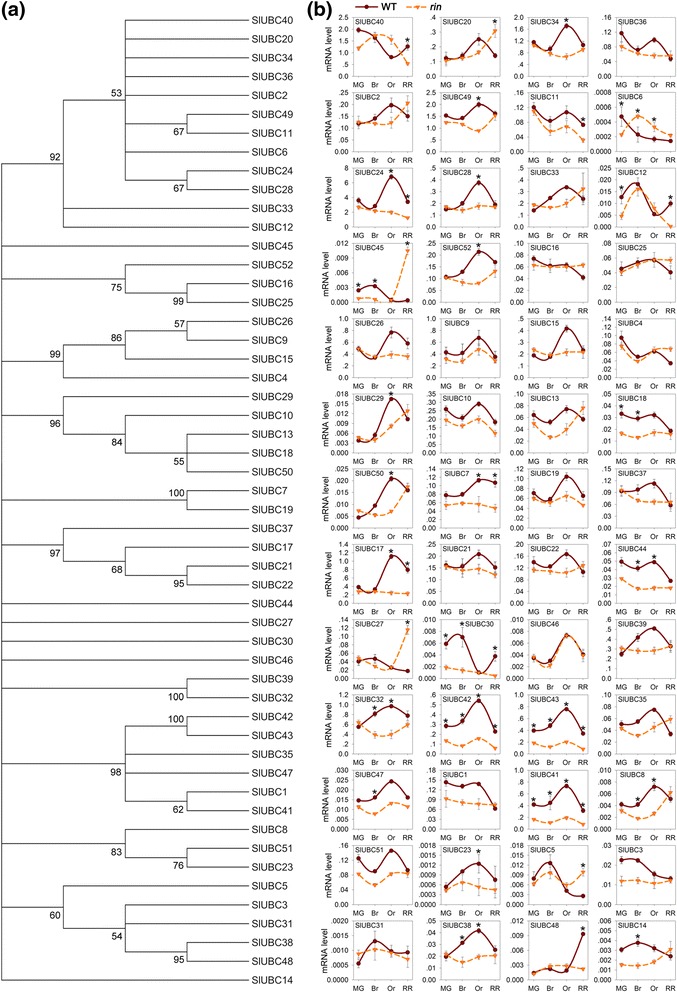
**Genome-wide screening and expression analysis of genes encoding E2 ubiquitin-conjugating enzymes in tomato. (a)** Phylogenetic analysis of tomato E2s. Phylogenetic tree was produced using MEGA version 5.2. Bootstrap values from 1,000 replications for each branch are shown. **(b)** Gene expression analysis of tomato E2s in wild-type (WT) and *rin* mutant during the period of fruit ripening, as determined by quantitative RT-PCR. The *actin* gene was used as the internal control. The stages of fruit ripening include mature green (MG), breaker (Br), orange (Or) and red ripe (RR). Values are means ± SD of three independent experiments. Asterisks indicate values that changed more than twofold in the *rin* mutant at indicated ripening stages.

Protein ubiquitination is mediated through the action of three enzymes known as ubiquitin-activating enzyme (E1), ubiquitin-conjugating enzyme (E2), and ubiquitin ligase (E3). Substrate specificity is mainly determined by E2 together with E3. In our quantitative analysis of nuclear proteome, we found that the expression of one ubiquitin E2 enzyme, SlUBC32, was downregulated in the *rin* mutant fruits. However, due to the limited sensitivity and resolution of proteomic technologies, the effect of RIN on the expression of other E2 remains unclear. By screening the SGN Tomato database, we identified 52 non-redundant E2 genes. These E2 genes were named *SlUBC1* to *SlUBC52* according to their location on the chromosomes (Additional file 8). All the E2 genes contain a highly conserved ubiquitin-conjugating (UBC) domain with an active-site cysteine residue confirmed by ScanProsite (Additional file 9). Phylogenetic analysis revealed that tomato ubiquitin E2 enzymes can be divided into a dozen of subgroups based on >50% bootstrap support (Figure 5a). Many tomato E2 proteins shared high similarity with another one (Additional file 10), suggesting gene duplications. The largest tomato E2 subgroup was formed by SlUBC2 and eleven other tomato E2s: SlUBC6, 11, 12, 20, 24, 28, 33, 34, 36, 40, and 49. SlUBC11, 20, 24, 28, 34, 36, 40, and 49 are very similar to SlUBC2, with more than or equal to 95% amino acid identity. SlUBC33 shows 91% identity to SlUBC2, while SlUBC6 and 12 show 87% and 84% identity to SlUBC2, respectively (Additional file 10). Gene expression of the 52 tomato E2 genes was analyzed in wild-type and *rin* mutant during fruit ripening using quantitative RT-PCR. Figure 5b shows the expression patterns of these genes as related to their phylogenetic relationships. Generally, gene expression patterns were frequently similar within subgroups. For example, the expression patterns of duplicated paralogs *SlUBC42* and *SlUBC43* showed high similarity. By contrast, the expression patterns of some paralogs, for example, *SlUBC20* and *SlUBC40*, were quite different. This suggests that, after duplication, one daughter gene may retain the ancestral function while the other acquires new function. We found that the expression of 14 E2 genes (*SlUBC7*, *8*, *12*, *17*, *18*, *24*, *30*, *32*, *38*, and *41* to *45*) was downregulated, whereas one (*SlUBC6*) was upregulated more than two-fold, in the *rin* mutant at two or more stages of ripening (Figure 5b).

### RIN binds directly to the promoter regions of five E2 genes *in vivo*

**Figure 6:**
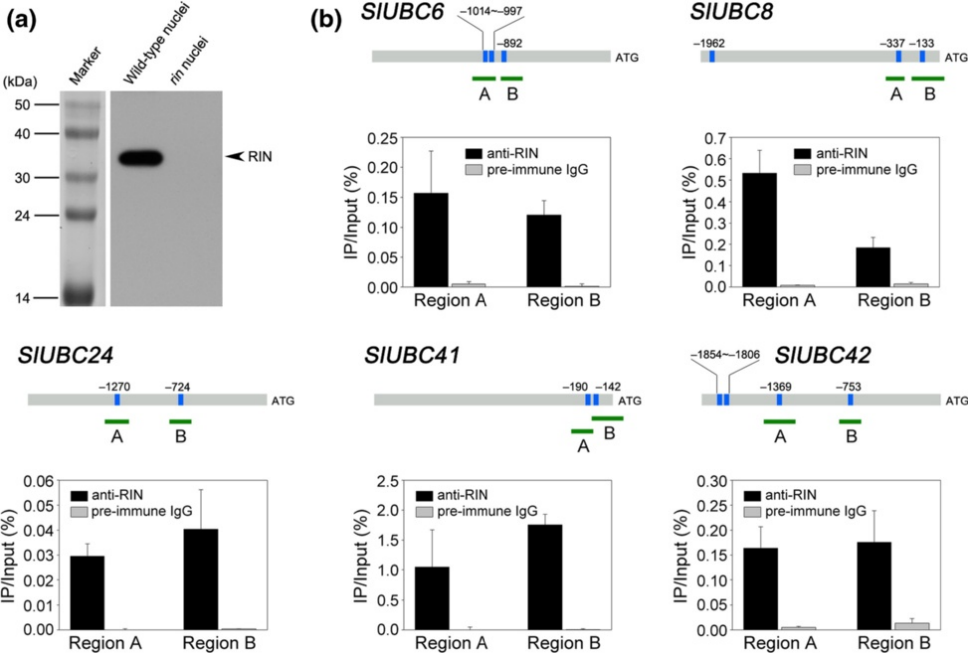
**RIN directly binds to the promoter regions of target genes as revealed by chromatin immunoprecipitation. (a)** Western blot revealed the specificity of the affinity-purified RIN polyclonal antibodies used for chromatin immunoprecipitation (ChIP) assay. Nuclear proteins were isolated from wild-type and *rin* mutant fruit at the orange ripening stage and hybridized with the RIN polyclonal antibodies. **(b)** ChIP-qPCR shows the binding of RIN to the promoter regions of five E2 genes. The promoter structures of the target genes are presented. Blue boxes represent CArG box elements and numbers indicate the position of these motifs relative to the translational start site. Green fragments with upper-case letters represent the regions used for ChIP-qPCR. Values are the percentage of DNA fragments that co-immunoprecipitated with anti-RIN antibodies (black bars) or non-specific antibodies (preimmune rabbit IgG; grey bars) relative to the input DNAs. Error bars represent the SD of three independent experiments.

Gene expression analysis showed that RIN affected the expression of 15 E2 genes at two or more stages of ripening (Figure 5b). Among these genes, *SlUBC32* has been identified as the direct target of RIN (Figure 4). To investigate whether RIN regulates the other 14 E2 genes by directly binding to their promoters, the ChIP assay was performed to probe DNA-protein interactions within the natural chromatin. Sequence analysis indicated that, except one (*SlUBC38*), 13 E2 genes contain CArG box binding motifs in their promoters (Additional file 5). For the ChIP assay, the affinity-purified anti-RIN polyclonal antibody (Figure 6a) was used to immunoprecipitate the cross-linked DNA-protein complexes. The enriched DNA was purified and then submitted to real-time quantitative PCR analysis. Primer sets were designed for those 13 genes that contain CArG box binding motifs in their promoters (Additional file 6). Our results indicate that RIN binds to the promoters of *SlUBC6*, *8*, *24*, *41*, and *42* (Figure 6b). Notably, RIN shows differential binding ability to the promoter fragments of these genes. The relative amounts of precipitated promoter fragments of *SlUBC6*, *24*, and *42* were low (<0.2%). By contrast, the relative amounts of precipitated promoter fragments of *SlUBC8* and *41* were much higher. The highest enrichments (>1%) was found for *SlUBC41* promoter fragments. Very low enrichments were observed for all fragments when cross-linked DNA-protein complexes were immunoprecipitated with pre-immune rabbit IgG, the non-specific antibody. This is considered as non-specific background enrichment.

### EMSA shows the *in vitro* binding activity of RIN

**Figure 7:**
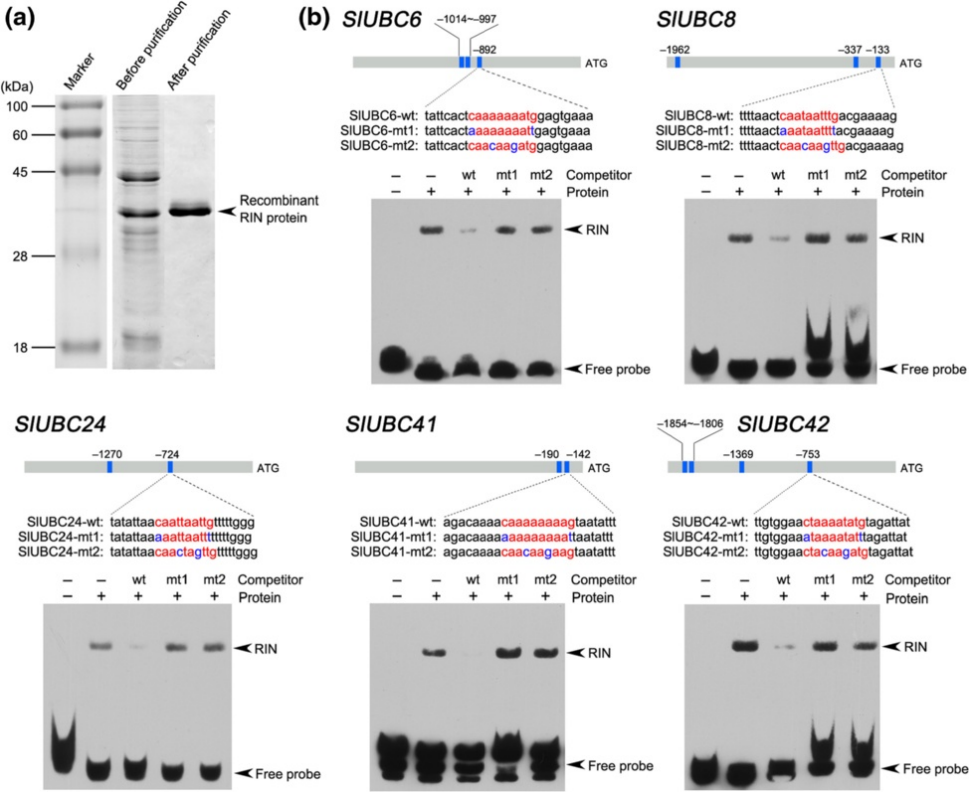
**Electrophoretic mobility shift assay of RIN binding to the regulatory regions of target genes. (a)** SDS-PAGE gel shows the affinity purification of the recombinant RIN protein used for the electrophoretic mobility shift assay. **(b)** RIN binds specifically to the promoters of target genes containing CArG box elements. The promoter structures of the target genes are presented. Blue boxes indicate CArG box elements in the promoter region and numbers represent the position of these motifs relative to the translational start site. The probe sequences for each target gene are shown, with red letters representing the CArG box. The mutated bases in the probes are represented by blue letters. wt, probe with intact CArG box element; mt, probe with mutated CArG box element. As competitors, 1,000-fold excess amounts of unlabeled probes were added to the binding reaction. The retarded bands and the free probes are indicated by arrowheads.

To confirm that RIN interacts with the promoters of E2 genes identified in the ChIP assay, we carried out an EMSA with purified recombinant RIN protein (Figure 7a). For each gene, a double-stranded and biotin-labeled probe (26-mer oligonucleotide) (Additional file 11) containing the CArG-box element was made, and its binding by the RIN protein was analyzed. A shift band was observed for each gene when the recombinant RIN protein was mixed with the biotin-labeled probe, indicating that RIN protein bound well to the biotin-labeled promoter fragments (Figure 7b). The binding of RIN protein to these fragments was effectively competed by addition of an excessive amount of the corresponding unlabeled probe with intact CArG box element, but not by the probe with mutated CArG box element. These results indicated that RIN binds specifically to the biotin-labeled probe. Furthermore, we observed different extents of competition by the unlabeled DNA fragment. This suggests that RIN has differential binding ability to the promoters of these genes. Together, our data suggest that RIN binds directly to the promoters of *SlUBC6*, *8*, *24*, *41*, and *42*. Considering *SlUBC32*, the direct RIN target identified on basis of the comparative analysis of nuclear proteome, a total of six E2 genes were identified as the direct targets of RIN.

### Specific E2 genes are involved in the regulation of fruit ripening

**Figure 8:**
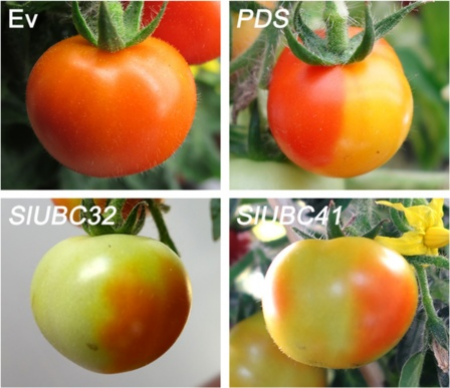
**Specific E2 genes are involved in the regulation of fruit ripening.** Virus-induced gene silencing assay in tomato reveals the effect of *SlUBC32* and *SlUBC41* on fruit ripening. Images show the ripe fruit of plants infected with vectors containing no insert (Ev), specific *PHYTOENE DESATURASE* sequence (*PDS*), specific *SlUBC32* sequence, or specific *SlUBC41* sequence.

To examine whether E2 genes participate in the regulation of fruit ripening, a virus-induced gene silencing (VIGS) assay was performed. All genes that were demonstrated to be directly regulated by RIN, namely *SlUBC6*, *8*, *24*, *32*, *41*, and *42*, were analyzed by this system. A specific cDNA fragment of these genes was cloned and inserted into the pTRV2 vector, respectively. The inflorescence peduncles at the preanthesis stage were used for infiltrated and the fruits were visually inspected daily. Obvious phenotype was detected in plants silenced for *SlUBC32* or *SlUBC41* cDNA. As shown in Figure 8, control fruit inoculated with pTRV2 alone (empty vector) showed a homogenous orange at the orange stage. By contrast, the color of fruits infected with pTRV2 carrying a 362 bp fragment of the *SlUBC32* gene was patchy with sectors of different shades of yellow and orange. Similar result was observed on fruits infected with the virus vector pTRV2 carrying a 477 bp fragment of the *SlUBC41* gene. The mRNA levels of *SlUBC32* and *SlUBC41* was measured by quantitative RT-PCR in the fruit pericarps of plants infiltrated with pTRV2-*SlUBC32* and pTRV2-*SlUBC41*. The results showed that the mRNA levels of *SlUBC32* and *SlUBC41* in the yellow areas were reduced by approximately 70% and 60%, respectively, when compared with the orange tissues. Inversely, the level of the TRV capsid protein mRNA was significantly higher in the yellow tissues (data not shown). These data demonstrated that *SlUBC32* and *SlUBC41* were successfully silenced and both genes play an important role in the regulation of tomato fruit ripening.

## Discussion

The nucleus is an important subcellular organelle that is essential for gene expression and regulation. We present here for the first time a comprehensive characterization of the nuclear sub-proteome of tomato fruits to seek the proteins involved in the regulation of fruit ripening. Sub-proteomic analysis enables the study of protein expression localized to a particular organelle, thereby providing additional insight into the protein function in a given condition [28-30]. The proteins associated with various cellular functions, for example, signaling, gene regulation, structure, proteolysis, detoxification, and translation were identified, implying the complexity of protein expression in the nucleus during fruit ripening. We additionally evaluated the changes of the nuclear proteome in the ripening-deficient mutant *rin*. We found that proteins involved in ubiquitin-proteasome system were regulated by RIN. Further study showed that RIN modulates protein ubiquitination by directly targeting specific E2 genes. Moreover, we provide evidence that two E2 genes are involved in the regulation of tomato fruit ripening.

### RIN directly targets genes involved in ubiquitin-proteasome pathway

The RIN transcription factor serves as one of the main ripening regulators. Characterization of the regulatory cascades controlled by RIN holds the promise to unravel the molecular regulatory mechanisms of fruit ripening. In our previous study, we identified 41 proteins representing 35 individual genes as potential targets of RIN through comparative proteomic analysis of total cellular proteins between wild-type and *rin* mutant tomato fruits [52]. We provided evidence that the regulatory effect of RIN on fruit ripening was partially achieved by targeting specific molecular pathways such as aroma formation [52], but it remained uncertain whether RIN regulates other molecular pathways. In the present study, we identified 127 proteins that changed abundance in the *rin* mutant by using an iTRAQ-based quantitative analysis of nuclear proteome. Except for S-adenosylmethionine synthase (Solyc01g101060 and Solyc09g008280), most of these proteins had not been recorded in our previous report [52]. Recently, Fujisawa *et al.* [53] reported the large-scale identification of direct RIN targets by chromatin immunoprecipitation coupled with DNA microarray analysis (ChIP-chip). More than two hundred of direct RIN target genes were identified that exhibit RIN-dependent positive or negative regulation during fruit ripening. The authors demonstrated that RIN participates in the regulation of lycopene accumulation, ethylene production, chlorophyll degradation, and many other physiological processes. However, possibly due to the lower sensitivity of ChIP-chip compared with ChIP-qPCR, less than half of the previously identified RIN targets were covered in this study, suggesting that some direct RIN targets still remain unidentified. By using nuclear proteome coupled with ChIP-qPCR and EMSA, we identify here two genes (*SlUBC32* and *PSMD2*) involved in ubiquitin-proteasome pathway as novel direct targets of RIN (Figure 4). Both *SlUBC32* and *PSMD2* are positively regulated by RIN (Figure 4a).

The ubiquitin-proteasome pathway is involved in the selective degradation of proteins in the cells of eukaryotic organisms. In this pathway, ubiquitin is attached to proteins destined for degradation and the resulting ubiquitin-protein conjugates are then recognized and catabolized by the 26S proteasome [54]. Three enzymes, that is, ubiquitin-activating enzymes (E1), ubiquitin-conjugating enzymes (E2), and ubiquitin ligases (E3) are responsible for the conjugation of ubiquitin to the substrate proteins [55]. *SlUBC32* that we identified as direct RIN target belongs to the E2 gene family. Numerous plant E2 subfamilies have been characterized biochemically [56,57], but relatively little is known about their functions, specificity, and regulation *in vivo*. Our study indicated that specific E2 was directly regulated by RIN. We also identified a protein (PSMD2) which is a component of the 26S proteasome as the direct RIN target. The 26S proteasome consisted of a 20S core particle containing multiple proteolytic sites and a 19S regulatory particle that directs the unfolded polypeptides into the core for breakdown [58]. It was demonstrated that the expression of the 26S proteasome subunit genes was regulated by a transcription factor Rpn4 in yeast [59]. However, the transcriptional regulation of 26S proteasome genes in plant remains largely unknown. In this study, we found that RIN directly bound the promoter of *PSMD2*, a gene encoding the 26S proteasome regulatory subunit, and regulated its expression. Notably, the expression of *SlUBC32* and *PSMD2* was only partially affected in the *rin* mutant fruits, suggesting that they are affected by other developmental factors in addition to RIN. Both *SlUBC32* and *PSMD2* were not identified in the ChIP-chip analysis reported by Fujisawa *et al.* [53], suggesting the value of sub-proteome analysis for identifying genes with crucial functions within a complex subcellular compartment.

### A set of E2 genes are identified as direct RIN targets

E2s are proteins capable of accepting ubiquitin from an E1 through a cysteine residue [60]. The resulting ubiquitin-E2 intermediate then delivers the ubiquitin to the substrate using an E3 as the recognition element. E2s contain a highly conserved region of approximately 140 to 150 amino acids called the ubiquitin-conjugating (UBC) domain that surrounds the active-site cysteine [57]. Because E2s are involved in both E3 selection and substrate modification, they function at the center of the ubiquitin transfer pathway and are responsible for much of the diversity of ubiquitin cellular signaling [61]. Thirty-seven E2 isoforms were identified in the Arabidopsis genome [62], and several of these E2 genes have been shown to play important roles in growth, development, and stress response [63,64]. By contrast, the family members of E2s in tomato and their biological functions remain largely uncharacterized.

In our quantitative analysis of nuclear proteome, we found that the expression of one ubiquitin E2 enzyme (SlUBC32) was downregulated in the *rin* mutant fruits. In order to determine whether other members of E2 family are also regulated by RIN, we firstly carried out an extensive search of the tomato genome to identify all potential E2s. Fifty-two E2s that contain a cysteine residue within a UBC domain were identified in tomato. We then examined the expression profiles of these E2s between wild-type and *rin* mutant tomatoes at different ripening stages. The data of quantitative RT-PCR indicated that, besides *SlUBC32* that we identified based on nuclear proteome, 14 E2s were differentially expressed in the *rin* mutant at two or more stages of tomato ripening (Figure 5b). Analysis of promoter regions of these genes showed that 13 of them contain CArG motifs, the typical binding sequence for RIN. To investigate whether these E2 genes are directly regulated by RIN, a ChIP assay was performed. Our results showed that RIN binds to the promoters of five E2 genes (*SlUBC6*, *8*, *24*, *41*, and *42*) *in vivo* (Figure 6). Further analysis using EMSA confirmed the results of ChIP analysis, showing that RIN bound to the promoters of these genes (Figure 7). Therefore, including the E2 gene that we identified based on nuclear proteome (*SlUBC32*), a total of six E2 genes were identified as the direct targets of RIN. All these E2 genes have not previous been identified as RIN direct targets. Among them, five (*SlUBC8*, *24*, *32*, *41*, and *42*) were positively regulated and one (*SlUBC6*) was negatively regulated by RIN. Our data indicated that specific E2 genes were regulated by RIN in a direct manner. Since E2s are required for the ubiquitin transfer pathway, it is conceivable that protein ubiquitination might be affected by RIN. As expected, we found that the ubiquitination levels of 53 proteins were altered in the nuclei of the *rin* mutant fruit by using SWATH-MS analysis (Additional file 7). Taken together, our results indicated that RIN regulated protein ubiquitination during fruit ripening by directly targeting specific E2 genes.

### Specific E2 genes are involved in the regulation of fruit ripening

Ubiquitin-mediated protein proteolysis plays an important role in many basic cellular processes in plants, such as cell cycle, circadian rhythm control, hormone signaling, growth, development, stress response, and disease resistance [54,63-66]. However, the functional importance of protein ubiquitination on fruit ripening remains to be determined. In our study, we demonstrated that a total of six E2 genes (*SlUBC6*, *8*, *24*, *32*, *41*, and *42*) were directly regulated by the tomato fruit-ripening regulator RIN, suggesting that specific E2 genes might be involved in fruit ripening. The function of these E2 genes was determined using the VIGS method. We found that silencing of either *SlUBC32* or *SlUBC41*, using specific fragments, resulted in altered fruit pigmentation at the orange ripening stage. This indicates that they are involved in the regulation of fruit ripening. Notably, the fruits of plants infiltrated with pTRV2-*SlUBC32* or pTRV2-*SlUBC41* turned homogenous red at the later stages of ripening. This suggests that homologous genes may exist to complement the function of *SlUBC32* and *SlUBC41*. Phylogenetic analysis indicated that *SlUBC32* is very similar to another E2 gene, *SlUBC39*, with 98.7% amino acid identity (Additional file 10). The expression patterns of *SlUBC32* and *SlUBC39* during tomato fruit ripening are also similar, suggesting gene duplications. Similar results were found for *SlUBC41*, which shows 74.8% and 72.2% identity to *SlUBC42* and *SlUBC43*, respectively. The expression patterns of *SlUBC41*, *SlUBC42*, and *SlUBC43* were similar in the process of fruit ripening. The study of the double or triple mutants, such as *Slubc32 Slubc39*, *Slubc41 Slubc42*, *Slubc41 Slubc43*, and *Slubc41 Slubc42 Slubc43*, will enable further understanding of the role of these genes in fruit ripening. The molecular mechanisms by which *SlUBC32* and *SlUBC41* regulate fruit ripening are currently unknown. Further studies are needed to determine the E3s that interacts with *SlUBC32* or *SlUBC41*, and their target proteins for ubiquitination.

## Conclusions

In summary, by quantitative proteome analysis of nuclear proteins isolated from tomato at various ripening stages, we identified a number of proteins that may play important roles in fruit ripening. Moreover, we investigated the changes in the nuclear proteome in the ripening-deficient mutant *rin*. Of the identified proteins, we focused on those involved in the ubiquitin-proteasome system. Gene expression analysis combined with ChIP assay and EMSA revealed that six genes encoding ubiquitin E2 enzymes are directly regulated by RIN. Further analysis using VIGS assays demonstrated that two E2s, *SlUBC32* and *SlUBC41*, are involved in the regulation of fruit ripening. To our knowledge, this is the first report for identifying specific E2s as regulators in fruit ripening. Our study unveils the novel function of protein ubiquitination and provides new insights into understanding the molecular regulatory network of fruit ripening.

## Material and methods

### Plant material

Seeds of wild-type tomato (*Solanum lycopersicum* cv Ailsa Craig) and ripening mutant *rin* in the cv Ailsa Craig background were kindly provided by Dr. James J. Giovannoni (Boyce Thompson Institute for Plant Research, Cornell University, Ithaca, NY, USA). Plants were grown in the greenhouse under standard culture conditions, with regular additions of fertilizer and supplementary lighting when required. Flowers were tagged at anthesis to accurately follow fruit ages through development. Fruits were harvested at mature green (MG), breaker (Br), orange (Or) and red ripe (RR), which were on average 42, 44, 46, and 48 days post anthesis (DPA), respectively. Ripening stages were defined based on the color, size, shape, seed development, and the development of locular jelly of the fruit [31]. Fruits of mutant *rin* were taken at the equivalent ripening stages as determined by the number of DPA. Immediately upon harvesting, pericarps were collected, frozen in liquid nitrogen, and stored at -80°C until use.

### Isolation of pure nuclei

Nuclei were isolated from tomato fruits following the method of Bowler *et al.* [67] with some modifications. All procedures were carried out on ice or at 4°C. Fruit samples were powdered in liquid nitrogen with a pestle and mortar and suspended in buffer 1 containing 0.4 M sucrose, 10 mM Tris-HCl, pH 8.0, 5 mM β-mercaptoethanol, and 1 mM PMSF. The cell debris in the homogenate was removed by filtering through four layers of sterile cheesecloth, then through two layers of miracloth (Calbiochem [68]). The homogenates were centrifuged at 3,000 × *g* for 10 min. The supernatants were discarded, and the pellets were gently resuspended in buffer 2 consisting of 0.25 M sucrose, 10 mM Tris-HCl, pH 8.0, 10 mM MgCl_2_, 1% Triton X-100, 5 mM β-mercaptoethanol, and 1 mM PMSF. After centrifugation at 10,000 × *g* for 10 min, the pellets were resuspended in 300 μL of buffer 3 containing 1.7 M sucrose, 10 mM Tris-HCl, pH 8.0, 0.15% Triton X-100, 2 mM MgCl_2_, 5 mM β-mercaptoethanol, and 1 mM PMSF. The resuspended pellets were then overlaid on top of 500 μL buffer 3 and centrifuged at 16,000 × *g* for 45 min. After centrifugation, the supernatants were removed and the enriched nuclei were collected. The degree of nuclei enrichment was evaluated by staining with DAPI and observed under a fluorescence microscope (Carl Zeiss [69]).

### Nuclear protein extraction and iTRAQ labeling

Nuclear proteins were isolated from the nuclei-enriched pellet using phenol extraction as previously described [70]. In brief, the nuclei were broken by sonification on ice in extraction buffer containing 0.7 M sucrose, 0.1 M KCl, 0.5 M Tris-HCl pH 7.5, 0.5 M EDTA, 1 mM PMSF, and 5 mM β-mercaptoethanol. Then an equal volume of Tris-HCl pH7.5-saturated phenol was added and the mixture was homogenized for 10 min. After centrifugation, the phenol phase in the upper layer was removed and re-extracted two times with extraction buffer. Proteins in the final phenol phase were precipitated overnight at -20°C with five volumes of ice-cold saturated ammonium acetate in methanol. The proteins were collected by centrifugation and washed with ice-cold methanol followed by multiple ice-cold acetone washes. Protein pellets were air-dried and stored at -80°C until use.

For proteomic analysis, the nuclear proteins were solubilized in protein buffer consisting of 500 mM triethylammonium bicarbonate (TEAB) and 0.6% SDS (w/v), pH 8.5. Protein concentrations were determined by the method of Bradford [71]. One hundred micrograms of proteins from each sample were reduced with 10 mM tris-(2-carboxyethyl) phosphine (TCEP), alkylated with 50 mM methyl methanethiosulfonate (MMTS), and digested with 10 ng μL^-1^ trypsin using the filter-aided sample preparation (FASP) method [72]. The tryptic peptides were then labeled with iTRAQ Reagents 4-plex Kit (Applied Biosystems [73]) according to the manufacturer’s protocol. Two separate iTRAQ experiments were carried out. For proteome analysis of different ripening stages, samples were taken from mature green, breaker, orange, and red ripe stages of wild-type and labeled with iTRAQ tags 114, 115, 116, and 117, respectively. For proteome analysis between wild-type and *rin* mutant, samples taken from wild-type and *rin* mutant at breaker stage were labeled with iTRAQ tags 114 and 115, respectively, while samples from wild-type and *rin* mutant at orange stage were labeled with iTRAQ tags 116 and 117, respectively. Two independent biological experiments with two technical replicates were performed. The iTRAQ-labeled samples were separately combined for the two set of experiments and then dried using a vacuum centrifuge. After reconstituted in 0.1% formic acid, 20 μL of the combined iTRAQ-labeled peptides were desalted using a C18 solid-phase extraction cartridge (Waters [74]) and submitted for NanoLC-MS/MS analysis.

### NanoLC-MS/MS analysis and protein identification

The MS analysis was performed using a NanoLC system (NanoLC-2D Ultra Plus, Eksigent [75]) equipped with a Triple TOF 5600 Plus mass spectrometer (AB SCIEX [76]). The iTRAQ-labeled peptide mixtures were desalted on a 100 μm × 20 mm trap column and then separated on an analytical 75 μm × 150 mm column. Both columns were filled with Magic C18-AQ 5 μm 200 Å phase (Michrom [77]). The mobile phase A was 0.1% formic acid in water, while mobile phase B was 0.1% formic acid in acetonitrile. Peptides were eluted in a linear gradient of 5-30% mobile phase B over 75 min at a flow rate of 300 nL/min. Precursor ions were selected across the mass range of 350 to 1500 m/z in high resolution mode (>30,000) using 250 ms accumulation time per spectrum. A maximum of 25 precursors per cycle from each MS spectrum were selected for fragmentation with 100 ms minimum accumulation time for each precursor and dynamic exclusion for 18 s. Tandem mass spectra were recorded in high sensitivity mode (resolution >15,000) with rolling collision energy and iTRAQ reagent collision energy adjustment on.

Protein identification and quantification for iTRAQ experiments was carried out using ProteinPilot™ 4.5 software (AB SCIEX). Database search was performed against the *Solanum lycopersicum* protein database ITAG2.4_proteins_full_desc.fasta with the following parameters: (1) Sample Type: iTRAQ 4-plex (Peptide Labeled); (2) Cysteine Alkylation: MMTS; (3) Digestion: Trypsin; (4) Instrument: TripleTOF 5600; (5) Species: *None*; (6) Quantitate: Yes; (7) Bias Correction: Yes; (8) Background Correction: Yes; (9) Search Effort: Thorough; (10) FDR Analysis: Yes. For iTRAQ quantitation, the peptide for quantification was automatically selected by the Pro Group™ algorithm (AB SCIEX) to calculate the reporter peak area. A reverse database search strategy was applied to estimate the global FDR for peptide identification. Only proteins identified below the 1% global FDR were ultimately exported for determining the meaningful cutoff value for the regulated proteins using a population statistics applied to the biological replicates [37]. Hierarchical clustering (Pearson algorithm) was performed with PermutMatrix software (version 1.9.3) [40].

### RNA isolation and quantitative RT-PCR analysis

RNA isolation from pericarp of the fruits was conducted using the method described by Moore *et al.* [78]. The extracted RNA was treated with DNase I (Promega [79]) and reverse transcribed with Moloney murine leukemia virus (M-MLV) reverse transcriptase (Promega).

Quantitative real-time PCR was performed with SYBR Green PCR Master Mix (Applied Biosystems) using the StepOne Plus Real-Time PCR System (Applied Biosystems). Gene-specific primers (Additional file 12) were designed with the Primer Express software 3.0 (Applied Biosystems). The following PCR program was used: 95°C for 10 min, followed by 40 cycles of 95°C for 15 s and 60°C for 30 s. Relative quantification of specific mRNA levels were measured using the cycle threshold (Ct) 2^(-ΔCt)^ method. Expression values were normalized using *actin* (SGN-U580609). Three independent biological replicates were analyzed for each sample.

### Preparation of RIN-specific antibody

For specific antibody preparation, a truncated form of *RIN* lacking the conserved MADS box was amplified from tomato cDNA using primers F (5′-TATAGGTACCGGTGAGGATTTGGGACAATTG-3′) and R (5′-TATAGGTACCCATTT GCTGTCCACCAGTC-3′) and inserted into the pET-30a vector. The plasmid was transformed into *Escherichia coli* BL 21 (DE3) competent cells. To express the recombinant protein, overnight culture of *E. coli* was diluted 1:100 in Luria Broth medium and incubated at 37°C until A_600_ reached approximately 0.5. Then 1 mM of isopropyl-1-thio-β-D-galactopyranoside (IPTG) was added to induce the expression of the recombinant protein. After incubated for an additional 3 h, the bacterial cells were collected. Recombinant protein was isolated from the bacterial cells and purified with Ni-NTA His Bind Resin following the manufacturer’s manual (Merck KGaA [80]). The recombinant protein was further purified by preparative gel electrophoresis. The protein band corresponding to RIN was excised from the gel and used to immunize rabbits at Beijing Protein Institute Co., Ltd. Polyclonal antibody that recognized RIN was affinity purified from antisera using AminoLink Plus Coupling Resin according to the purification protocol (Thermo Scientific [81]).

### Chromatin immunoprecipitation (ChIP)

The procedure for ChIP assay was modified from Bowler *et al.* [67]. Pericarp of the fruits was sliced and fixed with 1% formaldehyde under a vacuum, and then submitted to nuclear isolation as described above. Chromatin was sheared to an average length of approximately 500 to 1,000 bp by sonication. A small aliquot of sonicated chromatin was reversely cross-linked and served as the input DNA control. The remaining chromatin sample was centrifuged; the supernatant was diluted 10-fold in ChIP dilution buffer and pre-cleared using Protein A/agarose/salmon sperm DNA beads (Millipore [82]) for 1 h at 4°C. Immunoprecipitation was performed by incubating chromatin with affinity purified polyclonal anti-RIN antibody or pre-immune serum IgG (negative control) for 12 h at 4°C. The protein-chromatin immunocomplexes were captured using Protein A/agarose beads by incubating for 1 h at 4°C. The beads were collected and washed, and the immunocomplexes were eluted with elution buffer by gently rotating for 15 min at 65°C. Cross-linking of immunoprecipitated DNA was reversed by addition of NaCl to a final concentration of 0.2 M, and overnight incubation at 65°C. Proteins were digested with Proteinase K, and the immunoprecipitated DNA was purified using a QIAquick PCR Purification Kit (Qiagen [83]). Immunoprecipitated DNA was analyzed by real-time quantitative PCR using primers specific for the promoter regions of selected genes (Additional file 6).

### Electrophoretic mobility shift assay (EMSA)

The full-length *RIN* cDNA was amplified from tomato cDNA using primers RIN-F (5′-CGGGATCCATGGGTAGAGGGAAAGTAG-3′) and RIN-R (5′-CCGCTCGAGTCAAA GCATCCATCCAGGTAC-3′), digested with *BamH*I and *Xho*I, and cloned into the same restriction sites of pET-30a vector (Merck KGaA) to produce pET30a-RIN. This construct allows an in-frame fusion of the coding region of *RIN* with the N-terminal His-tag. The plasmid was transformed into *E. coli* BL 21 (DE3), and the recombinant protein expression and purification were performed as described above. EMSA was performed using the Lightshift Chemiluminescent EMSA kit (Thermo Scientific). Briefly, purified RIN protein in binding buffer containing 10 mM Tris-HCl, 50 mM KCl, 1 mM DTT, 2.5% glycerol, 0.05% NP-40, and 50 ng μL^-1^ polydeoxy (inosinate-cytidylate), pH 7.2 was incubated for 20 min at room temperature in the presence or absence of unlabeled (double-stranded) homologous or heterologous competitor probes. The 3′ biotin end-labeled double-stranded DNA probes, which were prepared by annealing complementary oligonucleotides, were then added, and the incubation was continued for 20 min. Native polyacrylamide gels (6%) were employed to separate protein-DNA complexes, and the biotin-labeled probes were detected according to the instructions provided by the EMSA kit. All oligonucleotide probes used in this study are listed in Additional file 11.

### Western blotting

For immunoblotting, proteins were separated by 12% SDS-PAGE and electrotransferred to Immobilon-P PVDF membrane (Millipore). The membranes were blocked for 2 h at room temperature with 5% BSA in PBS-Tween buffer (137 mM NaCl, 2.7 mM KCl, 8.1 mM NaH_2_PO_4_, 1.5 mM KH_2_PO_4_ and 0.1% Tween-20). Immunoblots were conducted overnight at 4°C. Antibodies used in this study included anti-Histone H3, anti-UDPase, anti-PsbA (AgriSera AB [84]), and anti-RIN. After washed with PBS-Tween (3 × 10 min), the membranes were treated with corresponding secondary antibodies conjugated to horseradish peroxidase. The immunoreactive bands were visualized by a chemiluminescence detection kit (SuperSignal®, Pierce Biotechnology [85]).

### Immunoprecipitation of ubiquitinated proteins for mass spectrometry

To enrich ubiquitinated proteins, the nuclei isolated from wild-type and *rin* mutant fruits at orange ripening stage were lysed by sonification on ice in IP buffer containing 50 mM Tris-HCl pH 7.5, 150 mM NaCl, 1% NP-40, 50 μM MG132, 1 mM PMSF, and the protease inhibitor cocktail tablet (Roch [86]). The lysate was centrifuged and the supernatant containing the ubiquitinated proteins were immunoprecipitated with 40 μL of Ub (P4D1)-agrose slurry (Santa Cruz [44]) overnight at 4°C. The agrose-beads were then collected in spin columns (Pierce Biotechnology) and washed with IP buffer twice. After elution from the beads with 0.1 M glycine-HCl (pH 2.2), the proteins were reduced, alkylated, and digested using the filter-aided sample preparation (FASP) method as describe above. The resulting peptides were collected, dried under vacuum, and redissolved with 0.1% formic acid for NanoLC-MS/MS analysis.

SWATH-MS was applied for quantitative analysis of ubiquitinated proteins between wild-type and *rin* mutant. SWATH-MS was conducted as previously described [46] with minor modifications. Data were acquired on a TripleTOF 5600 plus instrument (AB SCIEX) operating in SWATH mode. The same LC system and settings as for iTRAQ analysis described above were used. The MS1 spectra were collected in the range of 350 to 1,250 m/z with an accumulation time of 50 ms. The product ion MS/MS were collected in the range of 100 to 1,500 m/z with an accumulation time of 100 ms. Using an isolation width of 26 Da (containing 1 Da for the window overlap), a set of 32 overlapping windows was constructed covering the precursor mass range of 350 to 1,250 Da. The rolling collision energy and the high sensitivity mode were used. All of the data obtained were consolidated into a spectral library using ProteinPilot 4.5 software (AB SCIEX) and the *Solanum lycopersicum* protein database ITAG2.4_proteins_full_desc.fasta. The library was imported into Peakview software (AB SCIEX), which correlated both peptide identification and LC retention times to extract specific MS/MS transition data for each peptide with a confidence above 95%. The top five abundant ion transitions from the top ranked peptides for each protein were applied to retrieve quantitative data (in counts/s) using a 0.05 Da extraction width over a ±5 min LC time and visualized with MarkerView (AB SCIEX). For each individual sample, the same peptide transitions were summed into peptides, which were then summed into proteins. After normalizing using Total Area Sums, the extracted ions for selected proteins were analyzed using t-test within Markerview (AB SCIEX) with three technical replicates for each sample. A *P* value less than 0.05 was considered to be significant.

### Identification of tomato E2 family members

To identify members of the E2 gene family in tomato, the UBC domain of SlUBC32 obtained from Pfam (PF00179) [87] was used in BLAST searches against the Sol Genomics Network (SGN) tomato database [88]. The ScanProsite [89] and InterProScan [90] were applied to confirm the presence of the UBC domain. Sequences without an active-site cysteine residue in the UBC domain were excluded for further analysis. The uniqueness of the identified genes was manually verified and the redundant sequences were removed. The putative open reading frames (ORFs) and proteins sequences were predicted by GENSCAN [91].

### Phylogenetic analysis

The alignment of the protein sequences for tomato E2s (Additional file 13) was generated by ClustalX (version 2.1) software using default multiple parameters and PAM series protein weight matrix. Genedoc program was used to manually edit the alignment. The alignment was imported into MEGA (version 5.2) software and the phylogenetic tree was constructed using the neighbor-joining statistical method with 1,000 bootstrap replicates [92].

### Virus-induced gene silencing (VIGS)

VIGS assay was carried out following the method of Quadrana *et al.* [93]. The virus vectors pTRV1 and pTRV2 were kindly provided by Dr. Daqi Fu (College of Food Science and Nutritional Engineering, China Agricultural University, Beijing, China). The specific cDNA fragment corresponding to *SlUBC6*, *8*, *24*, *32*, *41*, and *42* were individually amplified, and inserted into the pMD19-T vector (TaKaRa Bio [94]). The plasmid was transformed into *E. coli*, and the insertion sequence was verified. The cDNA fragment was then cloned into the virus vector pTRV2 and subsequently transferred to *Agrobacterium tumefaciens* strain GV3101. The *phytoene desaturase* gene (*PDS*) served as the positive control. The *Agrobacterium* was grown at 28°C in Luria-Bertani medium supplemented with 50 mg L^-1^ gentamycin and 50 mg L^-1^ kanamycin. After harvested by centrifugation, the cells were resuspended in infiltration medium to obtain an optical density of 1.0 at 600 nm, and left at room temperature for 3 to 4 h. For plant inoculation, equivalent aliquots of *Agrobacterium* strain GV3101 containing pTRV1 or pTRV2 (empty or containing the insert) were mixed and injected to inflorescence peduncles of 8-week-old Micro-tom tomato plants.

### Data access

The mass spectrometry proteomics data have been deposited to the ProteomeXchange Consortium [95] via the PRIDE partner repository [96] with the dataset identifier PXD001414 [97].

## Additional files

Additional file 1:
**Workflow of the iTRAQ experiment for quantitative analysis of tomato nuclear proteome during fruit ripening.**
Additional file 2:
**Identification of the differentially expressed nuclear proteins during tomato fruit ripening using iTRAQ-based quantitative proteomic analysis.**
Additional file 3:
**Workflow of the iTRAQ experiment for quantitative analysis of tomato nuclear proteome in the** ***rin*** **mutant fruit.**
Additional file 4:
**Identification of the differentially expressed nuclear proteins in the** ***rin*** **mutant tomato fruit using iTRAQ-based quantitative proteomic analysis.**
Additional file 5:
**Predicted RIN binding motifs within 2,000 bp upstream region starting from ATG of genes analyzed in this study.**
Additional file 6:
**Primers used in the ChIP-qPCR analysis.**
Additional file 7:
**Identification of nuclear proteins that changed ubiquitination levels in the** ***rin*** **mutant tomato fruit using SWATH-MS approach.**
Additional file 8:
**The information of genes encoding E2 ubiquitin-conjugating enzymes in tomato.**
Additional file 9:
**Protein alignment of tomato E2 ubiquitin-conjugating enzymes (E2s) using Clustal X.** The protein sequences of all tomato E2s was used to generate the alignment. Only the core UBC domain is shown. Asterisk indicates the active-site cysteine residue within the UBC domain. Additional file 10:
**Similarities in the amino acid sequence of tomato E2 ubiquitin-conjugating enzymes.**
Additional file 11:
**Sequences of the probes used in EMSA.**
Additional file 12:
**Primers for quantitative RT-PCR analysis.**
Additional file 13:
**Protein sequences of tomato E2 ubiquitin-conjugating enzymes.**

## Abbreviations

ChIP: Chromatin immunoprecipitation
ChIP-chip: Chromatin immunoprecipitation coupled with DNA microarray analysis
DAPI: 4′,6-diamidino-2-phenylindole
DPA: Days post-anthesis
E1: Ubiquitin-activating enzyme
E2: Ubiquitin-conjugating enzyme
E3: Ubiquitin ligase
EMSA: Electrophoretic mobility shift assay
iTRAQ: Isobaric tags for relative and absolute quantification
PsbA: Photosystem II reaction centre protein D1
PSMD2: 26S proteasome regulatory subunit
quantitative RT-PCR: Quantitative reverse transcription polymerase chain reaction
*rin*: Ripening inhibitor
SlUBC32: Ubiquitin-conjugating enzyme E2 N
SWATH-MS: Single window acquisition of all theoretical spectra mass-spectrometry
UBC: Ubiquitin-conjugating
UDPase: UDP-glucose pyrophosphorylase
VIGS: Virus-induced gene silencing

## References

[1] Seymour GB, Østergaard L, Chapman NH, Knapp S, Martin C (2013). Fruit development and ripening. Annu Rev Plant Biol.

[2] Matas AJ, Gapper NE, Chung MY, Giovannoni JJ, Rose JK (2009). Biology and genetic engineering of fruit maturation
for enhanced quality and shelf-life. Curr Opin Biotechnol.

[3] Oeller PW, Lu MW, Taylor LP, Pike DA, Theologis A (1991). Reversible inhibition of tomato fruit senescence by
antisence RNA. Science.

[4] Hackett RM, Ho C, Lin Z, Foote HCC, Fray RG, Grierson D (2000). Antisense inhibition of the *Nr* gene restores normal ripening to the tomato *Never-ripe* mutant, consistent with the ethylene
receptor inhibition model. Plant Physiol.

[5] Barry CS, Giovannoni JJ (2006). Ripening in the tomato *Green-ripe* mutant is inhibited by ectopic expression of a protein
that disrupts ethylene signaling. Proc Natl Acad Sci U S A.

[6] Kevany BM, Tieman DM, Taylor MG, Cin VD, Klee HJ (2007). Ethylene receptor degradation controls the timing of
ripening in tomato fruit. Plant J.

[7] Guo H, Ecker JR (2004). The ethylene signaling pathway: new
insights. Curr Opin Plant Biol.

[8] Lin Z, Zhong S, Grierson D (2009). Recent advances in ethylene research. J Exp Bot.

[9] Merchante C, Alonso JM, Stepanova AN (2013). Ethylene signaling: simple ligand, complex
regulation. Curr Opin Plant Biol.

[10] Vrebalov J, Ruezinsky D, Padmanabhan V, White R, Medrano D, Drake R, Schuch W, Giovannoni J (2002). A MADS-box gene necessary for fruit ripening at the
tomato *Ripening-inhibitor* (*Rin*) locus. Science.

[11] Giovannoni JJ (2007). Fruit ripening mutants yield insights into ripening
control. Curr Opin Plant Biol.

[12] Manning K, Tor M, Poole M, Hong Y, Thompson AJ, King GJ, Giovannoni JJ, Seymour GB (2006). A naturally occurring epigenetic mutation in a gene
encoding an SBP-box transcription factor inhibits tomato fruit
ripening. Nat Genet.

[13] Lin Z, Hong Y, Yin M, Li C, Zhang K, Grierson D (2008). A tomato HD-Zip homeobox protein, LeHB-1, plays an
important role in floral organogenesis and ripening. Plant J.

[14] Itkin M, Seybold H, Breitel D, Rogachev I, Meir S, Aharoni A (2009). TOMATO AGAMOUS-LIKE 1 is a component of the fruit
ripening regulatory network. Plant J.

[15] Vrebalov J, Pan IL, Arroyo AJ, McQuinn R, Chung M, Poole M, Rose J, Seymour G, Grandillo S, Giovannoni J, Irish VF (2009). Fleshy fruit expansion and ripening are regulated by
the tomato *SHATTERPROOF* gene *TAGL1*. Plant Cell.

[16] Chung MY, Vrebalov J, Alba R, Lee J, McQuinn R, Chung JD, Klein P, Giovannoni J (2010). A tomato (*Solanum
lycopersicum*) *APETALA2/ERF* gene,
*SlAP2a*, is a negative regulator of fruit
ripening. Plant J.

[17] Karlova R, Rosin FM, Busscher-Lange J, Parapunova V, Do PT, Fernie AR, Fraser PD, Baxter C, Angenent GC, de Maagd RA (2011). Transcriptome and metabolite profiling show that
APETALA2a is a major regulator of tomato fruit ripening. Plant Cell.

[18] Bemer M, Karlova R, Ballester AR, Tikunov YM, Bovy AG, Wolters-Arts M, Rossetto Pde B, Angenent GC, de Maagd RA (2012). The tomato FRUITFULL homologs TDR4/FUL1 and MBP7/FUL2
regulate ethylene-independent aspects of fruit ripening. Plant Cell.

[19] Lee JM, Joung JG, McQuinn R, Chung MY, Fei Z, Tieman D, Klee H, Giovannoni J (2012). Combined transcriptome, genetic diversity and
metabolite profiling in tomato fruit reveals that the ethylene response factor
*SlERF6* plays an important role in ripening
and carotenoid accumulation. Plant J.

[20] Zhou T, Zhang H, Lai T, Qin C, Shi N, Wang H, Jin M, Zhong S, Fan Z, Liu Y, Wu Z, Jackson S, Giovannoni JJ, Rolin D, Gallusci P, Hong Y (2012). Virus-induced gene complementation reveals a
transcription factor network in modulation of tomato fruit
ripening. Sci Rep.

[21] Pan Y, Bradley G, Pyke K, Ball G, Lu C, Fray R, Marshall A, Jayasuta S, Baxter C, van Wijk R, Boyden L, Cade R, Chapman NH, Fraser PD, Hodgman C, Seymour GB (2013). Network inference analysis identifies an *APRR2-like* gene linked to pigment accumulation in
tomato and pepper fruits. Plant Physiol.

[22] Fujisawa M, Shima Y, Nakagawa H, Kitagawa M, Kimbara J, Nakano T, Kasumi T, Ito Y (2014). Transcriptional regulation of fruit ripening by tomato
FRUITFULL homologs and associated MADS box proteins. Plant Cell.

[23] Zhong S, Fei Z, Chen YR, Zheng Y, Huang M, Vrebalov J, McQuinn R, Gapper N, Liu B, Xiang J, Shao Y, Giovannoni JJ (2013). Single-base resolution methylomes of tomato fruit
development reveal epigenome modifications associated with
ripening. Nat Biotechnol.

[24] Bae MS, Cho EJ, Choi EY, Park OK (2003). Analysis of the *Arabidopsis* nuclear proteome and its response to cold
stress. Plant J.

[25] Choudhary MK, Basu D, Datta A, Chakraborty N, Chakraborty S (2009). Dehydration-responsive nuclear proteome of rice
(*Oryza sativa* L.) illustrates protein
network, novel regulators of cellular adaptation, and evolutionary
perspective. Mol Cell Proteomics.

[26] Pandey A, Chakraborty S, Datta A, Chakraborty N (2008). Proteomics approach to identify dehydration responsive
nuclear proteins from chickpea (*Cicer
arietinum* L.). Mol Cell Proteomics.

[27] Repetto O, Rogniaux H, Firnhaber C, Zuber H, Küster H, Larré C, Thompson R, Gallardo K (2008). Exploring the nuclear proteome of *Medicago truncatula* at the switch towards seed
filling. Plant J.

[28] Schirmer EC, Gerace L: **Organellar proteomics: the prizes and pitfalls of opening the nuclear envelope.** *Genome Biol* 2002, **3:**Reviews1008.1–1008.4. 10.1186/gb-2002-3-4-reviews1008 PMC139347 11983061

[29] Fink JL, Karunaratne S, Mittal A, Gardiner DM, Hamilton N, Mahony D, Kai C, Suzuki H, Hayashizaki Y, Teasdale RD (2008). Towards defining the nuclear proteome. Genome Biol.

[30] Oehring SC, Woodcroft BJ, Moes S, Wetzel J, Dietz O, Pulfer A, Dekiwadia C, Maeser P, Flueck C, Witmer K, Brancucci NM, Niederwieser I, Jenoe P, Ralph SA, Voss TS (2012). Organellar proteomics reveals hundreds of novel
nuclear proteins in the malaria parasite *Plasmodium
falciparum*. Genome Biol.

[31] Alba R, Payton P, Fei Z, McQuinn R, Debbie P, Martin GB, Tanksley SD, Giovannoni JJ (2005). Transcriptome and selected metabolite analyses reveal
multiple points of ethylene control during tomato fruit
development. Plant Cell.

[32] Osorio S, Alba R, Damasceno CM, Lopez-Casado G, Lohse M, Zanor MI, Tohge T, Usadel B, Rose JK, Fei Z, Giovannoni JJ, Fernie AR (2011). Systems biology of tomato fruit development: combined
transcript, protein, and metabolite analysis of tomato transcription factor
(*nor*, *rin*) and ethylene receptor (*Nr*)
mutants reveals novel regulatory interactions. Plant Physiol.

[33] Gygi SP, Rochon Y, Franza BR, Aebersold R (1999). Correlation between protein and mRNA abundance in
yeast. Mol Cell Biol.

[34] Chen G, Gharib TG, Huang CC, Taylor JM, Misek DE, Kardia SL, Giordano TJ, Iannettoni MD, Orringer MB, Hanash SM, Beer DG (2002). Discordant protein and mRNA expression in lung
adenocarcinomas. Mol Cell Proteomics.

[35] Lan P, Li W, Lin WD, Santi S, Schmidt W (2013). Mapping gene activity of Arabidopsis root
hairs. Genome Biol.

[36] Pradet-Balade B, Boulmé F, Beug H, Müllner EW, Garcia-Sanz JA (2001). Translation control: bridging the gap between genomics
and proteomics?. Trends Biochem Sci.

[37] Gan CS, Chong PK, Pham TK, Wright PC (2007). Technical, experimental, and biological variations in
isobaric tags for relative and absolute quantitation (iTRAQ). J Proteome Res.

[38] Ruepp A, Zollner A, Maier D, Albermann K, Hani J, Mokrejs M, Tetko I, Güldener U, Mannhaupt G, Münsterkötter M, Mewes HW (2004). The FunCat, a functional annotation scheme for
systematic classification of proteins from whole genomes. Nucleic Acids Res.

[39] **UniProt Knowledgebase.** [http://www.uniprot.org/]

[40] Caraux G, Pinloche S (2005). PermutMatrix: a graphical environment to arrange gene
expression profiles in optimal linear order. Bioinformatics.

[41] Bannister AJ, Kouzarides T (2011). Regulation of chromatin by histone
modifications. Cell Res.

[42] Ito Y, Kitagawa M, Ihashi N, Yabe K, Kimbara J, Yasuda J, Ito H, Inakuma T, Hiroi S, Kasumi T (2008). DNA-binding specificity, transcriptional activation
potential, and the *rin* mutation effect for
the tomato fruit-ripening regulator RIN. Plant J.

[43] **PLACE Web Signal Scan.** [http://www.dna.affrc.go.jp/PLACE/signalup.html]

[44] **Santa Cruz.** [http://www.scbt.com/]

[45] Pinato S, Gatti M, Scandiuzzi C, Confalonieri S, Penengo L (2011). UMI, a novel RNF168 ubiquitin binding domain involved
in the DNA damage signaling pathway. Mol Cell Biol.

[46] Gillet LC, Navarro P, Tate S, Röst H, Selevsek N, Reiter L, Bonner R, Aebersold R (2012). Targeted data extraction of the MS/MS spectra
generated by data-independent acquisition: a new concept for consistent and
accurate proteome analysis. Mol Cell Proteomics.

[47] Liu Y, Chen J, Sethi A, Li QK, Chen L, Collins B, Gillet LC, Wollscheid B, Zhang H, Aebersold R (2014). Glycoproteomic analysis of prostate cancer tissues by
SWATH mass spectrometry discovers N-acylethanolamine acid amidase and protein
tyrosine kinase 7 as signatures for tumor aggressiveness. Mol Cell Proteomics.

[48] Findlay GM, Smith MJ, Lanner F, Hsiung MS, Gish GD, Petsalaki E, Cockburn K, Kaneko T, Huang H, Bagshaw RD, Ketela T, Tucholska M, Taylor L, Bowtell DD, Moffat J, Ikura M, Li SS, Sidhu SS, Rossant J, Pawson T (2013). Interaction domains of Sos1/Grb2 are finely tuned for
cooperative control of embryonic stem cell fate. Cell.

[49] Saracco SA, Hansson M, Scalf M, Walker JM, Smith LM, Vierstra RD (2009). Tandem affinity purification and mass spectrometric
analysis of ubiquitylated proteins in Arabidopsis. Plant J.

[50] Kim DY, Scalf M, Smith LM, Vierstra RD (2013). Advanced proteomic analyses yield a deep catalog of
ubiquitylation targets in *Arabidopsis*. Plant Cell.

[51] Wang H, Wang L, Erdjument-Bromage H, Vidal M, Tempst P, Jones RS, Zhang Y (2004). Role of histone H2A ubiquitination in Polycomb
silencing. Nature.

[52] Qin G, Wang Y, Cao B, Wang W, Tian S (2012). Unraveling the regulatory network of the MADS box
transcription factor RIN in fruit ripening. Plant J.

[53] Fujisawa M, Nakano T, Shima Y, Ito Y (2013). A large-scale identification of direct targets of the
tomato MADS box transcription factor RIPENING INHIBITOR reveals the regulation
of fruit ripening. Plant Cell.

[54] Smalle J, Vierstra RD (2004). The ubiquitin 26S proteasome proteolytic
pathway. Annu Rev Plant Biol.

[55] Vierstra RD (2003). The ubiquitin/26S proteasome pathway, the complex last
chapter in the life of many plant proteins. Trends Plant Sci.

[56] Criqui MC, de Almeida EJ, Camasses A, Capron A, Parmentier Y, Inzé D, Genschik P (2002). Molecular characterization of plant
ubiquitin-conjugating enzymes belonging to the UbcP4/E2-C/UBCx/UbcH10 gene
family. Plant Physiol.

[57] Kraft E, Stone SL, Ma L, Su N, Gao Y, Lau OS, Deng XW, Callis J (2005). Genome analysis and functional characterization of the
E2 and RING-type E3 ligase ubiquitination enzymes of Arabidopsis. Plant Physiol.

[58] Clague MJ, Urbé S (2010). Ubiquitin: same molecule, different degradation
pathways. Cell.

[59] Xie Y, Varshavsky A (2001). RPN4 is a ligand, substrate, and transcriptional
regulator of the 26S proteasome: a negative feedback circuit. Proc Natl Acad Sci U S A.

[60] Glickman MH, Ciechanover A (2002). The ubiquitin-proteasome proteolytic pathway:
destruction for the sake of construction. Physiol Rev.

[61] Wenzel DM, Stoll KE, Klevit RE (2011). E2s: structurally economical and functionally
replete. Biochem J.

[62] Bachmair A, Novatchkova M, Potuschak T, Eisenhaber F (2001). Ubiquitylation in plants: a post-genomic look at a
post-translational modification. Trends Plant Sci.

[63] Xu L, Ménard R, Berr A, Fuchs J, Cognat V, Meyer D, Shen WH (2009). The E2 ubiquitin-conjugating enzymes, AtUBC1 and
AtUBC2, play redundant roles and are involved in activation of *FLC* expression and repression of flowering in
*Arabidopsis thaliana*. Plant J.

[64] Cui F, Liu L, Zhao Q, Zhang Z, Li Q, Lin B, Wu Y, Tang S, Xie Q (2012). *Arabidopsis* ubiquitin conjugase UBC32 is an
ERAD component that functions in brassinosteroid-mediated salt stress
tolerance. Plant Cell.

[65] Santner A, Estelle M (2010). The ubiquitin-proteasome system regulates plant
hormone signaling. Plant J.

[66] Park CH, Chen S, Shirsekar G, Zhou B, Khang CH, Songkumarn P, Afzal AJ, Ning Y, Wang R, Bellizzi M, Valent B, Wang GL (2012). The *Magnaporthe
oryzae* effector AvrPiz-t targets the RING E3 ubiquitin ligase APIP6
to suppress pathogen-associated molecular pattern-triggered immunity in
rice. Plant Cell.

[67] Bowler C, Benvenuto G, Laflamme P, Molino D, Probst AV, Tariq M, Paszkowski J (2004). Chromatin techniques for plant cells. Plant J.

[68] **Calbiochem.** [http://www.calbiochem.com/]

[69] **Carl Zeiss.** [http://www.zeiss.com/]

[70] Saravanan RS, Rose JK (2004). A critical evaluation of sample extraction techniques
for enhanced proteomic analysis of recalcitrant plant tissues. Proteomics.

[71] Bradford MM (1976). A rapid and sensitive method for the quantitation of
microgram quantities of protein utilizing the principle of protein-dye
binding. Anal Biochem.

[72] Wiśniewski JR, Zougman A, Nagaraj N, Mann M (2009). Universal sample preparation method for proteome
analysis. Nat Methods.

[73] **Applied Biosystems.** [http://www.appliedbiosystems.com/]

[74] **Waters.** [http://www.waters.com/]

[75] **Eksigent.** [http://www.eksigent.com/]

[76] **AB SCIEX.** [http://www.absciex.com/]

[77] **Michrom.** [http://www.dev.michrom.com/]

[78] Moore S, Payton P, Wright M, Tanksley S, Giovannoni J (2005). Utilization of tomato microarrays for comparative gene
expression analysis in the Solanaceae. J Exp Bot.

[79] **Promega.** [http://www.promega.com/]

[80] **Merck KGaA.** [http://www.merckgroup.com/]

[81] **Thermo Scientific.** [http://www.thermoscientific.com/]

[82] **Millipore.** [http://www.millipore.com/]

[83] **Qiagen.** [http://www.qiagen.com/]

[84] **AgriSera AB.** [http://www.agrisera.com/]

[85] **Pierce Biotechnology.** [http://www.piercenet.com/]

[86] **Roch.** [http://www.roche.com/]

[87] Punta M, Coggill PC, Eberhardt RY, Mistry J, Tate J, Boursnell C, Pang N, Forslund K, Ceric G, Clements J, Heger A, Holm L, Sonnhammer EL, Eddy SR, Bateman A, Finn RD: **The Pfam protein families database.** *Nucleic Acids Res* 2012, **40:**D290–D301. http://pfam.sanger.ac.uk. 10.1093/nar/gkr1065 PMC3245129 22127870

[88] **Sol Genomics Network (SGN) Tomato Database.** [http://solgenomics.net]

[89] **ScanProsite.** [http://www.expasy.ch/tools/scanprosite/]

[90] **InterProScan.** [http://www.ebi.ac.uk/tools/InterProScan/]

[91] **GENSCAN.** [http://genscanw.biosino.org/]

[92] Tamura K, Peterson D, Peterson N, Stecher G, Nei M, Kumar S (2011). MEGA5: Molecular evolutionary genetics analysis using
maximum likelihood, evolutionary distance, and maximum parsimony
methods. Mol Biol Evol.

[93] Quadrana L, Rodriguez MC, López M, Bermúdez L, Nunes-Nesi A, Fernie AR, Descalzo A, Asis R, Rossi M, Asurmendi S, Carrari F (2011). Coupling virus-induced gene silencing to exogenous
*green fluorescence protein* expression
provides a highly efficient system for functional genomics in Arabidopsis and
across all stages of tomato fruit development. Plant Physiol.

[94] **TaKaRa Bio.** [http://www.takara-bio.co.jp/]

[95] Vizcaíno JA, Deutsch EW, Wang R, Csordas A, Reisinger F, Ríos D, Dianes JA, Sun Z, Farrah T, Bandeira N, Binz PA, Xenarios I, Eisenacher M, Mayer G, Gatto L, Campos A, Chalkley RJ, Kraus HJ, Albar JP, Martinez-Bartolomé S, Apweiler R, Omenn GS, Martens L, Jones AR, Hermjakob H (2014). ProteomeXchange provides globally co-ordinated
proteomics data submission and dissemination. Nat Biotechnol.

[96] Vizcaino JA, Côté RG, Csordas A, Dianes JA, Fabregat A, Foster JM, Griss J, Alpi E, Birim M, Contell J, O’Kelly G, Schoenegger A, Ovelleiro D, Pérez-Riverol Y, Reisinger F, Ríos D, Wang R, Hermjakob H (2013). The Proteomics IDEntifications (PRIDE) database and
associated tools: status in 2013. Nucleic Acids Res.

[97] **ProteomeXchange.** [http://www.ebi.ac.uk/pride]

