# Supplementary material for: Tomato nuclear proteome reveals the involvement of specific E2 ubiquitin-conjugating enzymes in fruit ripening
Source: Genome Biol. 2014 Dec 3;15(12):548. doi: 10.1186/s13059-014-0548-2 (PMC4269173; doi:10.1186/s13059-014-0548-2)
Supplement: Additional file 1: — Workflow of the iTRAQ experiment for quantitative analysis of tomato nuclear proteome during fruit ripening. [file 13059_2014_548_MOESM1_ESM.pdf]

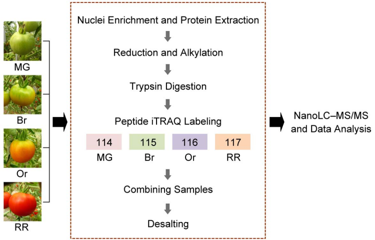

Additional file 1. Workflow of the iTRAQ experiment for quantitative analysis of tomato nuclear proteome during fruit ripening.
