# Supplementary material for: Tomato nuclear proteome reveals the involvement of specific E2 ubiquitin-conjugating enzymes in fruit ripening
Source: Genome Biol. 2014 Dec 3;15(12):548. doi: 10.1186/s13059-014-0548-2 (PMC4269173; doi:10.1186/s13059-014-0548-2)
Supplement: Additional file 5: — Predicted RIN binding motifs within 2,000 bp upstream region starting from ATG of genes analyzed in this study. [file 13059_2014_548_MOESM5_ESM.pdf]

Additional file 5. Predicted RIN binding motifs within 2000-bp upstream region starting from ATG of genes analyzed in this study.

Gene	ITAG gene ID[a]	SGN unigene[b]	Position	CArG box	Motif number
PSMD2	Solyc07g053650	SGN-U569045	-197	CTAATTTTTG	4
			-622	CAAATATATG	
			-669	CTTAAAATTG	
			-681	CCATAAATGG	
SlUBC6	Solyc02g083570	SGN-U565334	-829	CAAAAAAATG	3
			-997	CAAATAATAG	
			-1014	CATATATATG	
SlUBC7	Solyc02g084760	SGN-U579325	-238	CTTTAATTAG	2
			-916	CATATATAAG	
SlUBC8	Solyc02g085690	—	-133	CAATAATTTG	3
			-337	CAATAATTTG	
			-1926	CTTTAAAAAG	
SlUBC12	Solyc03g033410	SGN-U565335	-1106	CAATAAATTG	2
			-1892	CTAATATTAG	
SlUBC17	Solyc04g011430	SGN-U593391	-321	CAAAAATATG	5
			-453	CAAAAATTAG	
			-484	CATATTTTTG	
			-1067	CATAAAATAG	
			-1800	CAAAAAATAG	
SlUBC18	Solyc04g078620	SGN-U592148	-184	CAAATAATTG	3
			-272	CAATATTAAG	
			-1520	CTAATATTTG	
SlUBC24	Solyc06g007510	SGN-U578218	-724	CAATTAATTG	2
			-1210	CAAAAATTTG	
SlUBC30	Solyc07g024070	SGN-U568707	-1759	CAAAAAATAG	3
			-1801	CATTATAAAG	
			-1926	CTAAAATATG	
SlUBC32	Solyc07g062570	SGN-U576994	-502	CAAAAAATAG	4
			-852	CATAATTATG	
			-1347	CTAATAATAG	
			-1827	CATATATATG	
SlUBC38	Solyc10g007000	SGN-U567052	—	—	0

SlUBC41	Solyc10g012240	—	-142	CAAAAAAAAG	2
			-190	CAATTTAATG	
SlUBC42	Solyc10g012270	—	-753	CTAAAATATG	4
			-1369	CATTTTTAAG	
			-1806	CAAAAAAAAG	
			-1854	CAATTTAATG	
SlUBC43	Solyc10g012320	—	-1339	CATTTATTTG	1
SlUBC44	Solyc10g081160	SGN-U571107	-4	CTTATATTTG	1
SlUBC45	Solyc11g065190	SGN-U581052	-1406	CATAAAAATG	2
			-1892	CATTAATTTG	
ACS2	Solyc01g095080	SGN-U567978	-371	CATTTAAAAG	3
			-497	CTTAAATTTG	
			-1682	CTAAAAAAAG	

[a]ITAG, the International Tomato Annotation Group release version 2.3.
[b]SGN identification number of the best BLAST hit in the Sol Genomics Network (SGN) tomato unigene database (http://solgenomics.net).
