# Supplementary material for: Tomato nuclear proteome reveals the involvement of specific E2 ubiquitin-conjugating enzymes in fruit ripening
Source: Genome Biol. 2014 Dec 3;15(12):548. doi: 10.1186/s13059-014-0548-2 (PMC4269173; doi:10.1186/s13059-014-0548-2)
Supplement: Additional file 6: — Primers used in the ChIP-qPCR analysis. [file 13059_2014_548_MOESM6_ESM.pdf]

Additional file 6. Primers used in the ChIP-qPCR analysis.

Gene	ITAG gene ID[a]	SGN unigene[b]	Position	Forward primer (5'–3')	Reverse primer (5'–3')
PSMD2	Solyc07g053650	SGN-U569045	-197	AAGGTTCCTTCTAAGGACCCAAAG	ATCCTCCTCCGACTAATCCCAGCA
			-622	GCCCATTCTTTACTTACCAGATCC	GAACAAAGGTTTTCGTCTTCACCA
SlUBC6	Solyc02g083570	SGN-U565334	-829	CAAATTAGGCAAATGGTACAGAGA	GGACGGCATACATTATTCAAATAT
			-997	GGCAGCGTAAAAAACTGTTACA	TTCCACTATTATTTGCTGTTACCAT
SlUBC7	Solyc02g084760	SGN-U579325	-238	TGCTGTCCAACTTTTACGTACAA	TCCACCTTCTACTTCCCTTATTTAG
			-916	TCCATTACTTGATATTTGTTTGG	TCGATTCGGATAAGTTTTACCCA
SlUBC8	Solyc02g085690	—	-133	GCATCCTCGTGACATTTAATATTT	TGGGCTTTTTTGAGTTCTATTCT
			-337	TGATACACGTGCAATTTAACAACT	CTCAAAACTATCCTTCCCATCAA
SlUBC12	Solyc03g033410	SGN-U565335	-1106	GCTATCTTTTAGGTGCATACAGGAT	AGCTCGTCATATCAGGTTTGATT
			-1892	GCAAACTTGATTGGCACTCA	CCACTTCACAATCATTTTCATTAAC
SlUBC17	Solyc04g011430	SGN-U580887	-321	TTTTAAACGGCAAAGACATTATA	TTTGGAACCATTTGTGGAAGAGT
			-453	TAACCATGAACATACTTCCCTCA	ATCGGTGGATCAAATTATGACAC
SlUBC18	Solyc04g078620	SGN-U592148	-184	TTGAATATTTATATTCAATGA	CACTCTTCAGCAGGACGAACAAA
			-272	TCATGCACTTTTCTCAATAATAC	ATTCTTTTTAGTTTTATC
SlUBC24	Solyc06g007510	SGN-U578218	-724	TGACGAATGAAGGAGATCTAGTTT	CTTACTCTCAATCCCAAATTAGCA
			-1210	TGAGCTAAAGGCAAAAATTTGTA	CGTATCCAAAAACTGATCAATTG
SlUBC30	Solyc07g024070	SGN-U568707	-1759	AAACGAAAAATATATT	TTGCACTTATTTCAATATAT
			-1801	AAATAACATAGAATAGAAAAA	AATATATTTTTCGTTTCCTTTA

SlUBC32	Solyc07g062570	SGN-U576994	-502	ACGCGCGTCAATTGAGGTTA	CATTTTCCCCTCTTTTTCTTGAAGA
			-852	TCTTAGGGACCATAAATGAACGG	TGCCTTCAAATAATTGCGATGCA
SlUBC41	Solyc10g012240	SGN-U271269	-142	TCAAGTAACTATGTTACATAAATAA	AAAGAATCAGTTGCTTGTACAAC
			-190	TTATAGCTTTATAAATATCTACAAC	TTTATTTATGTAACATAGTTACTTG
SlUBC42	Solyc10g012270	—	-753	CTCCGTGTTGGTCGTAGTATTAGT	GGTGAGATTACCTACACGCAATAA
			-1369	CCTGCTGAAAGTAAATCTCCTCTT	CCCTTCAAGTTACAAAAAAAACAC
SlUBC43	Solyc10g012320	—	-1339	CATTCACCTTGCGTACATTGTT	GAACCCGTATCTGCATCATTATAA
SlUBC44	Solyc10g081160	SGN-U571107	-4	CAGGATTCTTTCAGAAGCTTGATTT	CATAGCCAAATATAAGTTGATGTCC
SlUBC45	Solyc11g065190	SGN-U581052	-1406	CCGAGCCTGACGAGAATTT	GGCCATTTCCATCAACAAAA
			-1892	CGCTCAATTGATGCATACATG	CACACCCTTACTAGGCTTCAAA
ACS2	Solyc01g095080	SGN-U567978	-1682	TCACAAACGAGCTATTCTAAAAA	CCTTACATCATTTATTATTACAA

[a]ITAG, the International Tomato Annotation Group release version 2.3.
[b]SGN identification number of the best BLAST hit in the Sol Genomics Network (SGN) tomato unigene database (http://solgenomics.net).
