# Supplementary material for: Tomato nuclear proteome reveals the involvement of specific E2 ubiquitin-conjugating enzymes in fruit ripening
Source: Genome Biol. 2014 Dec 3;15(12):548. doi: 10.1186/s13059-014-0548-2 (PMC4269173; doi:10.1186/s13059-014-0548-2)
Supplement: Additional file 8: — The information of genes encoding E2 ubiquitin-conjugating enzymes in tomato. [file 13059_2014_548_MOESM8_ESM.pdf]

Additional file 8. The information of genes encoding E2 ubiquitin-conjugating enzymes in tomato.

Gene	ITAG gene ID[a]	SGN unigene[b]	Size (AA)	pI	Mw (kD)
SlUBC1	Solyc01g094810	SGN-U579048	184	4.24	20.8
SlUBC2	Solyc01g095490	SGN-U580883	148	8.06	16.5
SlUBC3	Solyc01g111680	SGN-U604257	911	4.32	100.7
SlUBC4	Solyc02g067420	SGN-U579926	152	5.74	17.3
SlUBC5	Solyc02g078210	SGN-U567723	925	4.56	102.8
SlUBC6	Solyc02g083570	SGN-U565334	148	8.33	16.7
SlUBC7	Solyc02g084760	SGN-U579325	161	7.54	18.3
SlUBC8	Solyc02g085690	—	226	8.75	25.7
SlUBC9	Solyc02g087750	SGN-U580405	152	5.20	17.3
SlUBC10	Solyc02g093110	SGN-U592742	160	7.99	18.1
SlUBC11	Solyc03g007470	SGN-U579276	148	8.06	16.6
SlUBC12	Solyc03g033410	SGN-U565335	150	8.48	16.9
SlUBC13	Solyc03g044260	SGN-U577380	160	8.33	17.9
SlUBC14	Solyc03g112720	SGN-U596593	147	11.05	16.7
SlUBC15	Solyc03g113100	SGN-U580749	152	5.20	17.3
SlUBC16	Solyc03g123660	SGN-U583412	239	9.11	26.9
SlUBC17	Solyc04g011430	SGN-U593391	165	4.85	18.6
SlUBC18	Solyc04g078620	SGN-U592148	160	7.56	18.0
SlUBC19	Solyc04g080810	SGN-U580669	161	8.33	18.4
SlUBC20	Solyc05g050230	SGN-U581187	148	8.06	16.4
SlUBC21	Solyc05g054540	SGN-U579417	170	4.83	18.9

SlUBC22	Solyc05g054550	SGN-U578313	170	4.81	19.0
SlUBC23	Solyc06g007500	—	262	4.98	30.4
SlUBC24	Solyc06g007510	SGN-U578218	148	8.06	16.5
SlUBC25	Solyc06g063100	SGN-U583410	229	7.98	25.9
SlUBC26	Solyc06g070980	SGN-U578489	152	5.20	17.3
SlUBC27	Solyc06g072570	SGN-U582847	194	4.73	21.4
SlUBC28	Solyc06g082600	SGN-U578566	148	8.06	16.5
SlUBC29	Solyc07g021660	—	138	5.58	16.3
SlUBC30	Solyc07g024070	SGN-U568707	295	9.54	33.1
SlUBC31	Solyc07g053960	SGN-U589396	302	3.93	33.4
SlUBC32	Solyc07g062570	SGN-U576994	153	7.51	17.2
SlUBC33	Solyc07g066080	SGN-U568401	148	8.47	16.4
SlUBC34	Solyc08g008220	SGN-U580936	148	8.06	16.5
SlUBC35	Solyc08g081270	SGN-U562770	183	4.21	20.9
SlUBC36	Solyc08g081950	SGN-U580479	119	8.22	13.5
SlUBC37	Solyc09g009720	SGN-U586931	167	4.98	18.8
SlUBC38	Solyc10g007000	SGN-U567052	667	7.11	72.8
SlUBC39	Solyc10g007260	SGN-U576995	153	7.51	17.2
SlUBC40	Solyc10g011740	SGN-U578152	148	8.06	16.5
SlUBC41	Solyc10g012240	—	183	4.21	20.8
SlUBC42	Solyc10g012270	—	127	6.07	14.6
SlUBC43	Solyc10g012320	—	134	6.22	15.6
SlUBC44	Solyc10g081160	SGN-U571107	263	9.33	28.5
SlUBC45	Solyc11g065190	SGN-U581052	181	4.94	20.0
SlUBC46	Solyc11g071260	SGN-U563892	157	8.62	17.7

SlUBC47	Solyc11g071870	SGN-U571404	185	4.15	21.1
SlUBC48	Solyc12g013820	SGN-U587749	742	7.06	81.4
SlUBC49	Solyc12g056100	SGN-U578242	148	8.08	16.5
SlUBC50	Solyc12g088680	SGN-U274770	161	8.47	18.1
SlUBC51	Solyc12g089030	SGN-U577494	183	6.50	20.8
SlUBC52	Solyc12g099310	SGN-U569767	301	4.94	34.1

[a]ITAG, the International Tomato Annotation Group release version 2.3.

[b]SGN identification number of the best BLAST hit in the Sol Genomics Network (SGN) tomato unigene database (http://solgenomics.net).
