# Supplementary material for: Tomato nuclear proteome reveals the involvement of specific E2 ubiquitin-conjugating enzymes in fruit ripening
Source: Genome Biol. 2014 Dec 3;15(12):548. doi: 10.1186/s13059-014-0548-2 (PMC4269173; doi:10.1186/s13059-014-0548-2)
Supplement: Additional file 9: — Protein alignment of tomato E2 ubiquitin-conjugating enzymes (E2s) using Clustal X. The protein sequences of all tomato E2s was used to generate the alignment. Only the core UBC domain is shown. Asterisk indicates the active-site cysteine residue within the UBC domain. [file 13059_2014_548_MOESM9_ESM.pdf]

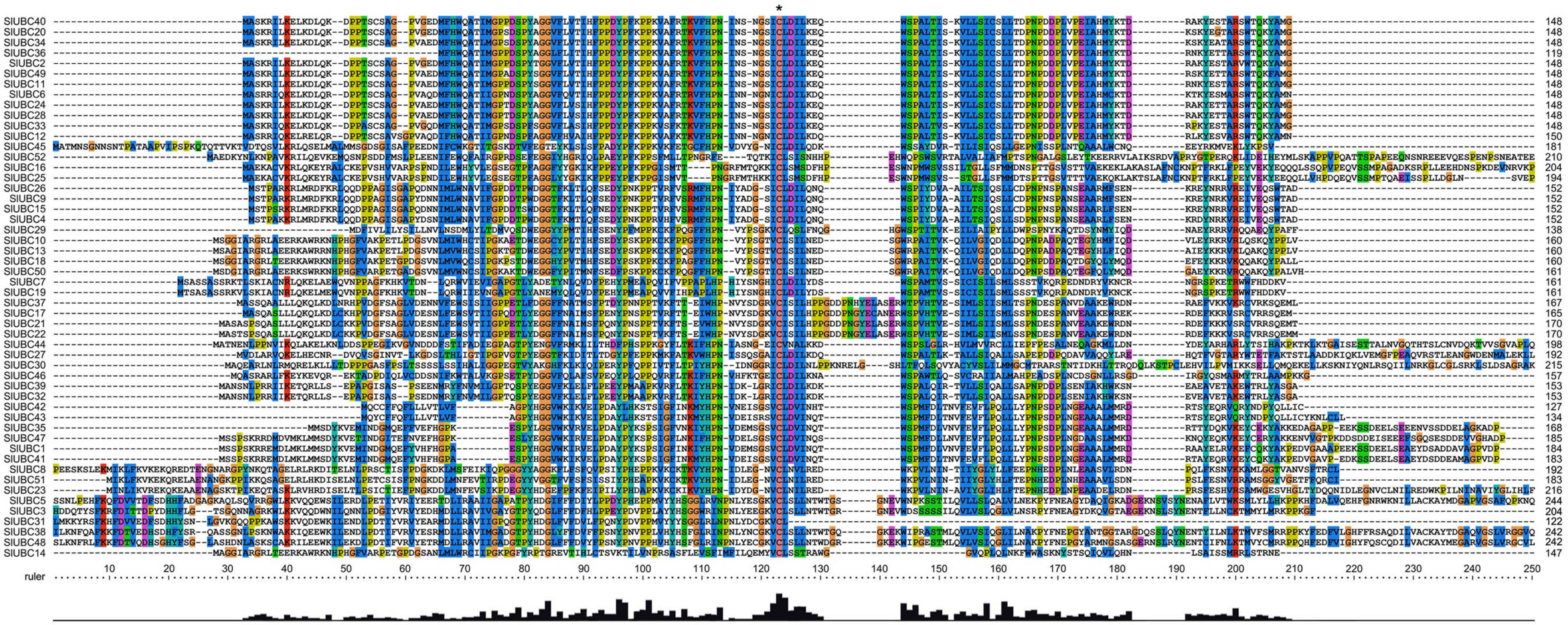

Additional file 9. Protein alignment of tomato E2 ubiquitin-conjugating enzymes (E2s) using Clustal X. The amino acids of all tomato E2s was used to generate the alignment. Only the core UBC domain is shown. Asterisk indicates the active-site cysteine residue within the UBC domain.
