# Supplementary material for: Tomato nuclear proteome reveals the involvement of specific E2 ubiquitin-conjugating enzymes in fruit ripening
Source: Genome Biol. 2014 Dec 3;15(12):548. doi: 10.1186/s13059-014-0548-2 (PMC4269173; doi:10.1186/s13059-014-0548-2)
Supplement: Additional file 11: — Sequences of the probes used in EMSA. [file 13059_2014_548_MOESM11_ESM.pdf]

Additional file 11. Sequences of the probes used in EMSA.

Gene	ITAG Gene ID[a]	SGN unigene[b]	Probes	Sequence (5'–3')[c]
PSMD2	Solyc07g053650	SGN-U569045	PSMD2-wt	TTTTGTTACTAATTTTTGAGGGATAT
			PSMD2-mt1	TTTTGTTAATAATTTTTTAGGGATAT
			PSMD2-mt2	TTTTGTTACTACTTGTTGAGGGATAT
SlUBC6	Solyc02g083570	SGN-U565334	SlUBC6-wt	TATTCACTCAAAAAAATGGAGTGAAA
			SlUBC6-mt1	TATTCACTAAAAAAAATTGAGTGAAA
			SlUBC6-mt2	TATTCACTCAACAAGATGGAGTGAAA
SlUBC8	Solyc02g085690	—	SlUBC8-wt	TTTTAACTCAATAATTTGACGAAAAG
			SlUBC8-mt1	TTTTAACTAAATAATTTTACGAAAAG
			SlUBC8-mt2	TTTTAACTCAACAAGTTGACGAAAAG
SlUBC24	Solyc06g007510	SGN-U578218	SlUBC24-wt	TATATTAACAATTAATTGTTTTTGGG
			SlUBC24-mt1	TATATTAAAAATTAATTTTTTTTGGG
			SlUBC24-mt2	TATATTAACAACTAGTTGTTTTTGGG
SlUBC32	Solyc07g062570	SGN-U576994	SlUBC32-wt	AAAGTAAACAAAAAATAGTTACGAAG
			SlUBC32-mt1	AAAGTAAAAAAAAAATATTTACGAAG
			SlUBC32-mt2	AAAGTAAACAACAAGTAGTTACGAAG
SlUBC41	Solyc10g012240	—	SlUBC41-wt	AGACAAAACAAAAAAAAGTAATATTT
			SlUBC41-mt1	AGACAAAAAAAAAAAAATTAATATTT
			SlUBC41-mt2	AGACAAAACAACAAGAAGTAATATTT
SlUBC42	Solyc10g012270	—	SlUBC42-wt	TTGTGGAACTAAAATATGTAGATTAT
			SlUBC42-mt1	TTGTGGAAATAAAATATTTAGATTAT
			SlUBC42-mt2	TTGTGGAACTACAAGATGTAGATTAT

[a]ITAG, the International Tomato Annotation Group release version 2.3.

[b]SGN identification number of the best BLAST hit in the Sol Genomics Network (SGN) tomato unigene database (http://solgenomics.net).

[c]The CArG-box elements were marked in red. The mutated bases in the probes are represented by blue letters. wt, probe with intact CArG box element; mt, probe with mutated CArG box element.
