# Supplementary material for: Tomato nuclear proteome reveals the involvement of specific E2 ubiquitin-conjugating enzymes in fruit ripening
Source: Genome Biol. 2014 Dec 3;15(12):548. doi: 10.1186/s13059-014-0548-2 (PMC4269173; doi:10.1186/s13059-014-0548-2)
Supplement: Additional file 12: — Primers for quantitative RT-PCR analysis. [file 13059_2014_548_MOESM12_ESM.pdf]

Additional file 12. Primers for quantitative RT-PCR analysis.

Gene	ITAG gene ID[a]	SGN unigene[b]	Primers	Sequence (5'–3')
PSMD2	Solyc07g053650	SGN-U569045	Forward<br>Reverse	GCTGGACTGGTTACTCTGCT<br>ACCTGCCTGACCTACAACAT
SlUBC1	Solyc01g094810	SGN-U579048	Forward<br>Reverse	TGCCCCTGAGGAGAAGTCAA<br>CAGGGCCTGCAACTGCAT
SlUBC2	Solyc01g095490	SGN-U580883	Forward<br>Reverse	TGCCCCTGAGGAGAAGTCAA<br>CAGGGCCTGCAACTGCA
SlUBC3	Solyc01g111680	SGN-U603899	Forward<br>Reverse	GAGTACCAACATCTCCACCAATTG<br>CTGACCTTGGCTGGATTGAAC
SlUBC4	Solyc02g067420	SGN-U579926	Forward<br>Reverse	GGAAGGTGCGTGAAATAGTTGAG<br>CCAGGAGAAAGCCATGAAATG
SlUBC5	Solyc02g078210	SGN-U567723	Forward<br>Reverse	CCCAAGAACCAGGACAAAGAAC<br>CTCCACGAGTTTAGGGTAAAGCTT
SlUBC6	Solyc02g083570	SGN-U565334	Forward<br>Reverse	TCCACTGGTTCCAGAAATTGC<br>TTTGAGTCCAACTACGAGCCATT
SlUBC7	Solyc02g084760	SGN-U579325	Forward<br>Reverse	ATGTTGTCAAGTTCAACCGTGAA<br>TGGGAGATCTGCCGTTTCTG
SlUBC8	Solyc02g085690	—	Forward<br>Reverse	GAGGCTGCTTCTGTGTTGAGAGA<br>GCCACAGTTCCACCAAGCAT
SlUBC9	Solyc02g087750	SGN-U580405	Forward<br>Reverse	GAAGGTGCGTGAGATTGTTGAG<br>CAGCATGGAGAACATTGCAGTT
SlUBC10	Solyc02g093110	SGN-U580409	Forward<br>Reverse	GCCAAACCCTGCTGATCCT<br>CGCACCCTCTTTCGGTACTC
SlUBC11	Solyc03g007470	SGN-U579276	Forward<br>Reverse	GATCCTTTGGTCCCGGAGAT<br>TGGGTCCAGCTCCTTGCA
SlUBC12	Solyc03g033410	SGN-U565335	Forward<br>Reverse	CAGACGATCCATTGGTACCAGAA<br>CCAACTACGAGCCACTGATTCA
SlUBC13	Solyc03g044260	SGN-U577380	Forward<br>Reverse	TCCGCCTCTGGTGTAGTCTTAATA<br>GCCAAAACAGTGTCAGCTATCAGT
SlUBC14	Solyc03g113100	SGN-U596593	Forward<br>Reverse	CCCGGATTTCTTTTCTTACACCTA<br>TCTGTTGGGCAATATGCATAGATG
SlUBC15	Solyc03g113100	SGN-U580749	Forward<br>Reverse	TCAGAAGCTGCTCGGTTGTTT<br>TGTCCAGCTCTGCTCCACAAT
SlUBC16	Solyc03g123660	SGN-U583412	Forward<br>Reverse	CAACAGAGGAAATCCGTTCCA<br>AGCAGAGGCAGGGCCATTA
SlUBC17	Solyc04g011430	SGN-U580887	Forward<br>Reverse	GCATCATATCCATGCTTTCAAGTC<br>CCTTTTTTCCCTCCATTCCTTAG

SlUBC18	Solyc04g078620	SGN-U592148	Forward<br>Reverse	CGCACAAACCGATGGCTAT<br>ACTGCTTAGCCTGCTGCTTCA
SlUBC19	Solyc04g080810	SGN-U580669	Forward<br>Reverse	CTGCGGACAACGATCGCTAT<br>ATCGTCATGGAACCACCATCTC
SlUBC20	Solyc05g050230	SGN-U581187	Forward<br>Reverse	CAGTGGAGCCCTGCATTAACTA<br>CGGTTCCTTCGTATTTGGACTT
SlUBC21	Solyc05g054540	SGN-U579417	Forward<br>Reverse	GCTAATGTCGAAGCAGCTAAGGA<br>GGGACCGTCTTACACAACGACTA
SlUBC22	Solyc05g054550	SGN-U578313	Forward<br>Reverse	GCTAATGTCGAAGCAGCTAAGGA<br>CTTGAGACCGTCTTACACAACGA
SlUBC23	Solyc06g007500	—	Forward<br>Reverse	GAAGATCCACTTAATCATGAAGCA<br>CCCCACATCGTCCTCCTAAC
SlUBC24	Solyc06g007510	SGN-U578218	Forward<br>Reverse	ACTGACAGGGCCAAATACGAA<br>GGAGACAATTTTGCGCATCA
SlUBC25	Solyc06g063100	SGN-U583410	Forward<br>Reverse	CATGGCAGAAAAGGCATGTGT<br>CCACAACATGGGAGACAGGTT
SlUBC26	Solyc06g070980	SGN-U578489	Forward<br>Reverse	GGAAGCAGCTCGGATGTTCA<br>CAGTCCAGCTCTGCTCAACAAT
SlUBC27	Solyc06g072570	SGN-U582847	Forward<br>Reverse	GGAGTACTTTGGAAGCAAATGG<br>ATCAGCTTTTGCTGGAAGGA
SlUBC28	Solyc06g082600	SGN-U578566	Forward<br>Reverse	AAGACTGACAGGGCCAAATACG<br>CGAGGACAATGCCGTTATCC
SlUBC29	Solyc07g021660	—	Forward<br>Reverse	TGGGTGGAGTCCTACCATTACA<br>TATAATTAGGGCTCGGCCAAT
SlUBC30	Solyc07g024070	SGN-U568707	Forward<br>Reverse	GGTCCCAGATCATTTTGTGAA<br>TGCACGTCCAGCAGAGTCTA
SlUBC31	Solyc07g053960	SGN-U589396	Forward<br>Reverse	GGAAGGAAGAGGTGGCTGAA<br>CGAGAAGTCCTCTACCACATCAA
SlUBC32	Solyc07g062570	SGN-U576994	Forward<br>Reverse	TGGACACGCCTATATGCTAGTG<br>GAGGGTGCTATCCAGGTTCA
SlUBC33	Solyc07g066080	SGN-U568401	Forward<br>Reverse	AATACGCAATGGGTTGAACACA<br>TGTGCACCATGCCTAAGTTTATTT
SlUBC34	Solyc08g008220	SGN-U580936	Forward<br>Reverse	CTGGACCCAAAAGTATGCAATG<br>CCACAAACTCAGAGGGAAAGGA
SlUBC35	Solyc08g081270	SGN-U562770	Forward<br>Reverse	TGCGAGAAATACGCTAAGAAAGAA<br>TTTTCCAGCAAGTTCATCATCACT
SlUBC36	Solyc08g081950	SGN-U580479	Forward<br>Reverse	GGTTAGTTGCTGTTGCAATGCA<br>CCCCTTGAAACTGATTAAGCCATA
SlUBC37	Solyc09g009720	SGN-U586931	Forward	CGAATGTTGATGCGGCTAAG

			Reverse	TGATTTCCTCACGCATCTCTTC
SlUBC38	Solyc10g007000	SGN-U567052	Forward	AGGAGATTGGCGTAAAGGATTG
			Reverse	TTGCAGGAACAACTGGTGCTA
SlUBC39	Solyc10g007260	SGN-U576995	Forward	CAAAGCACTGGAAGTCAAACGA
			Reverse	TCAGGCACCACTTGCATAAAGA
SlUBC40	Solyc10g011740	SGN-U578152	Forward	CAAACCCCGATGATCCTTTG
			Reverse	CCGGGCTGTTGATTCGTATT
SlUBC41	Solyc10g012240	SGN-U271269	Forward	CCGGAGGAGAAGTCGAGTGA
			Reverse	TCAACAGGGCCTGCCATT
SlUBC42	Solyc10g012270	—	Forward	CTCCCACAACTTCTCTTGTATCCT
			Reverse	GTTCGGTCTCGCATCATCAG
SlUBC43	Solyc10g012320	—	Forward	CTCCCACAACTTCTCTTGTATCCT
			Reverse	GTTCGGTCTCGCATCATCAG
SlUBC44	Solyc10g081160	SGN-U571107	Forward	GAAGATCGACGCGAGAAAGAA
			Reverse	TCTCCCATCTATCAAACAAAAGCA
SlUBC45	Solyc11g065190	SGN-U581052	Forward	CAAACATAAGCTCACCTCTGAACA
			Reverse	GCTTCTCAACCATCTTCCTGTATT
SlUBC46	Solyc11g071260	SGN-U563892	Forward	CGATCCGGTGATATCAGAGGTT
			Reverse	TTTTAGCCCTTCTTGGGCATT
SlUBC47	Solyc11g071870	SGN-U571404	Forward	CGCAAAGAAGGAGAATGTCGTT
			Reverse	TCTGTCCACTGAACTCCTCTTCAC
SlUBC48	Solyc12g013820	SGN-U587749	Forward	CGCCGTCTAGATACTTCTCTCAGA
			Reverse	TTCCGGCACCTGCACATT
SlUBC49	Solyc12g056100	SGN-U578242	Forward	ACTCAAAAATTTGCCATGGGATA
			Reverse	GGAATCAGACCCCGATGGT
SlUBC50	Solyc12g088680	SGN-U274770	Forward	GTGATCCAGCACAAACAGAAGGT
			Reverse	GGCCTGCTGTCTCACTCGTT
SlUBC51	Solyc12g089030	SGN-U577494	Forward	GACAACCCCAGCTTGTTCGA
			Reverse	CAGCGTTGGAAGAACGTTTCT
SlUBC52	Solyc12g099310	SGN-U569767	Forward	ATCTAAAAAATCCAGCGGTGAAGA
			Reverse	GCTCATGAAATCATCGGAAGGA
Actin	Solyc11g005330	SGN-U580609	Forward	TGTCCCTATTTACGAGGGTTATGC
			Reverse	AGTTAAATCACGACCAGCAAGAT

[a]ITAG, the International Tomato Annotation Group release version 2.3.

[b]SGN identification number of the best BLAST hit in the Sol Genomics Network (SGN) tomato unigene database (http://solgenomics.net).
