# Supplementary material for: Tomato nuclear proteome reveals the involvement of specific E2 ubiquitin-conjugating enzymes in fruit ripening
Source: Genome Biol. 2014 Dec 3;15(12):548. doi: 10.1186/s13059-014-0548-2 (PMC4269173; doi:10.1186/s13059-014-0548-2)
Supplement: Additional file 13: — Protein sequences of tomato E2 ubiquitin-conjugating enzymes. [file 13059_2014_548_MOESM13_ESM.pdf]

Additional file 13. Protein sequences of tomato E2 ubiquitin-conjugating enzymes.

>SlUBC1
MSSPSKRREMDLMKLMMSDYKVEMINDGMQEFYVHFHGPAESPYHGGVWKIRVELPDAYPY
KSPSIGFINKIYHPNVDEMSGSVCLDVINQTWSPMFDLVNVFEVFLPQLLLYPNPSDPLNGEAAA
LMMRDRAAYELRVKEFCQKYAKPEDVGAAAPEEKSSDEELSEAEYDSADDAVAGPVDP

>SlUBC2
MASKRILKELKDLQKDPPTSCSAGPVGEDMFHWQATIMGPPDSPYTGGVFLVTIHFPPDYPFKP
PKVAFRTKVFHPNINSNGSICLDILKEQWSPALTISKVLLSICSLLTDPNPDDPLVPEIAHMYKTDRA
KYESTARVWTQKYAMG

>SlUBC3
MDESESTESINNVIVVDRGFLHGDYVAAASDPTGQVGLVVDINISVDLLAHDGSIFKDVSSRELKR
VRGFTVGDYVVLGPWLGRIDDVFDNVTVMFDDGSVCKVMKADPLRLKPVGRDGLEDGHFPFY
PGQRVKASSSSVFKNSRWLSGSWKANRLEGTVTKVTVGSVFIYWIASAGYGPDSSTAPAEEQNP
KNLKLMSCFSHAIWQLGDWCLLPSSFALDKQLSKLQLSDSTKTVSESSQPLTDGDSEVVHLEEST
GNSDCMEIDVESSVDGNCETLEHDYLAESSTCANSLSLSKESGQESWPLHRKKIRKVVVRRDKKA
RKKEENFERALLIVNTRTSVDVAWQDGKIEGGLESTSLIPIESPGDHEFVAEQYVVEKAADDADDS
NDVRRVGVVKSVNAKERTASVRWLKLVTRAEDPKEFDKEEVVSVYELEGHPDYDYCYGDVVVRL
LPVSLPAKVGSVLTSTEESEHLLVPVEAKEDEQKHSKCNEAEAAPSDDTCSQFSDLSWVGNITGLR
NGDIEVTWADGMISLVGPQAIYVVDRDDDESIVAGSDVGDDVASWETVEDHERETLGNVEEEL
GTTNATDISIEDEDGAMATEDSGRNGALSIPLAALGFVTRLASGIFSRGRKQTDSSSLDSRSEDEE
REGTFAKIFTGDDSWSQRSGDLDNSPRLPAAGNAEDHDTMEVTDVIEANLTSEMGNSSDQHD
DQTYSFKRFDITTDPYDHHFLGTSGQNNAGRKWLKKVQQDWNILQNNLPDGIYVRVYEDHMD
LLRAVIVGAYGTPYQDGLFFFDFHLPPEYPDVPPLAYYHSGGWRINPNLYEEGKVCLSLLNTWTG
RGNEVWDSSSSSILQVLVSLQGLVLNSRPYFNEAGYDKQVGTAEGEKNSLSYNENTFLLNCKTM
MYLMRKPPKGF

>SlUBC4
MSTPAKKRLMRDFKRLQQDPPAGISGAPYDSNIMLWNAVIFGPDDTPWDGGTFKMTLQFSED
YPNKPPRVHFVSRMFHPNIYADGSICLDILQNQWSPIYDVAAILTSIQSLLSDPNPNSPANSEAAR
LFSENKRDYNRKVREIVEQSWTAD

>SlUBC5
MDTSLSDFDSFSESSSYDDQDYVEYLYGGHACSILSSLEESIGKIDDFLSFERVFMYGDIVCSEKEPS
GQMGKVVNVEMTVDLECIYGSKIQDVNSKDLVKIRPISVGDYVVMGPWLGKVEKIVDKIKVLFD
DGAKSEFSAEASEILTPISPDLVEDPQFPFYPGQRVQVQSVSASGSTSWLCGVRSGKREQGTIYAV
EAGVVHVDWIGCGSLGCEKMPSPPTLQDSEKLTLLSCYSHAKWQLGDCCVLPVADSKNIVRKSI
QSSPPCGPMEQDRQLNKASQKTNRSSTFLQVAVISKTRTKVDILWQDGSVTTGLDSDSVFPVNI
VDAHEFWPEHFVLEKGMGDDSSVPSPKRWGVVRCVDAKERTVKVKWTTYSLHEPNNFRVEQS
EEIVSAYELMDHPDYSYCLGDAVCKFCEDQVFSLEGKSLSTHMFSETGMDSNTDLKNVDTGKDN
LDFPKYDHLSCIGIIVGFKDGDIEIKWATGFTSMVAPFEIYRIDKCEAAVSISASNAENAEPSNVEM
SSNESQLSKHEEKGLLKFGSNSESCNESLWDSGSCLISRTAVGFFSSITSTLFGSLSISLFGTYQTISEE

GQKSRIVDEEEVIELSHLNAGIPTFENLKASPEMELEQVQETTEGQKDDALPSSSNLPEHFKQFDV
VTDFSDHHFADGAGKAQLSQVRRGWLKKVQQEWSILERDLPETIYVRIYEERTDLIRAAIIGAPAT
PYHDGIFFFDIYLPPDYPHEPPMVYYHSGGLRVNPNLYESGKVCLSLLNTWTGSGNEVWNPKSS
TILQVLLSLQALVLNEKPYFNEAGYDAQIGKADGEKNSVSYNENAFLVTWKSMLYLLHKPPKHFD
ALVQEHFGNRWKNILLACKAYMDGAPVGSAFQPKNQDKEPIKGSSTGFKIMLGKLYPKLVEAFS
NKGIDCSQLSD

>SlUBC6
MASRRIQKELKDLQRDPPTSCSAGPVAQDMFHWQATIIGPNDSPYAGGVFQVTIHFPPDYPFKP
PKVAFRTRVFHPNINNNGNICLDILKDQWSPALTISKVLLSICSLLTDPNPDDPLVPEIAHMCKTDK
TKYESMARSWTQKYAMN

>SlUBC7
MSASSASSRKTLSKIACNRLQKELAEWQVNPPAGFKHKVTDNLQRWVIEVIGAPGTLYADETYN
LQVDFPEHYPMEAPQVIFVPPAPLHPHIYSNGHICLDILYDSWSPAMTVSSICISILSMLSSSTVKQ
RPEDNDRYVKNCRNGRSPKETRWWFHDDKV

>SlUBC8
MVCYANPCFLSHSIYTNNFSSLLSPLKVFFIQFSPEESKSLEKMIKLFKVKEKQREDTENGNARGPY
NKQTAGELRLRKDITELNLPRSCTISFPDGKDKLMSFEIKIQPGGGYYAGGKFLFSFQVPSIYPHEP
PKVKCKTKVYHPNIDLEGNVCLNVLREDWKPVLNINTIIYGLYLLFEEPNHEDPLNLEAASVLRDN
PQLFKSNVKKAMLGGTVANVSFTRCL

>SlUBC9
MSTPARKRLMRDFKRLQQDPPAGISGAPYDNNIMLWNAVIFGPDDTPWDGGTFKLTLQFSEDY
PNKPPTVRFVSRMFHPNIYADGSICLDILQNQWSPIYDVAAILTSIQSLLCDPNPNSPANSEAARM
FSENKREYNRKVREIVEQSWTAD

>SlUBC10
MSGGIARGRLAEERKAWRKNHPHGFVAKPETLPDGSVNLMIWHCTIPGKAETDWEGGCYPLTI
HFSEDYPSKPPKCKFPPGFFHPNVYPSGTVCLSILNEDSGWRPAITVKQILVGIQDLLDQPNPADP
AQTEGYHMFIQDVLEYRKRVRLQSKQYPPLV

>SlUBC11
MASKRILKELKDLQKDPPTSCSAGPVAEDMFHWQATIMGPPDSPYSGGVFLVTIHFPPDYPFKP
PKVAFRTKVFHPNINSNGSICLDILKEQWSPALTISKVLLSICSLLTDPNPDDPLVPEIAHMYKTDRN
KYESTARSWTQKYAMG

>SlUBC12
MASRRIQKELRELQRDPPTSCSAVSGPVAQDIFHWQATIIGPNDSPFAGGVFHVAIHFPPDYPFK
PPKVAFKTKVFHPNINNNGNICLDILKDQWSPALTISKVLLSICSLLTDPNPDDPLVPEIAHMYKTD
RLKYESVARSWTQKYAMN

>SlUBC13

MSAGIARGRLAEERKAWRRNHPHGFVAKPETLPDGSVNLMVWHCSIPGKTGTDWEGGCYPV
TIHFSEDYPSKPPKCKFPQGFFHPNVYPSGTVCLSILNEDSGWRPAITVKQILVGIQDLLDQPNPA
DPAQTEGYHLFIQDAIEYKKRVKLQAKQYPPLV

>SlUBC14
MAGGIARGRLTEERKAWRKNHPHGFVARPETGPDGSANLMLWRCIIPGKPGFYRPTGREVTIH
LCTSVKTILVNPRSASFLEVSFIMFILQEMYVCLSSTRAWGGVQPLQLNKFWWASKNYSTSQIQV
LQHNLSAISSMRRLSTRNE

>SlUBC15
MSTPSRKRLMRDFKRLQQDPPAGISGAPQDNNIMLWNAVIFGPDDTSWDGGTFKLTLQFNED
YPNKPPTVRFVSRMFHPNIYADGSICLDILQNQWSPIYDVAAILTSIQSLLCDPNPNSPANSEAARL
FSENKREYNRRVREIVEQSWTAD

>SlUBC16
MAEKACVKRLQKEYRALCKEPVSHVVARPSPNDILEWHYVLEGSEGTPFAGGFYYGKIKFPPEYP
FKPPGISMTTPNGRFMTQKKICLSMSDFHPESWNPMWSVSSILTGLLSFMMDNSPTTGSVSTT
VAEKEKLAKASLAFNCKNPTFRKLFPEYVEKYEQQQLSSQPVPEQVSSMPAGADKSRPLLEEHD
NSPKDEVNRVKPLKDVKNQQRKSVPTWLLLLLVSIFGVVMALPLLQL

>SlUBC17
MASQASLLLQKQLKDLCKHPVDGFSAGLVDESNLFEWSVTIIGPQDTLYEGGFFNAIMSFPENYP
NSPPTVRFTTEIWHPNVYSDGKVCISILHPPGDDPNGYECANERWSPVHTVESIILSIISMLSSPN
DESPANVEAAKEWREKRDEFKKRVSRCVRRSQEM

>SlUBC18
MSGGIARGRLTEERKAWRKNHPHGFVAKPETGPDGSVNLMVWQCSIPGKPSTDWEGGHYPV
TMHFSEDYPSKPPKCKFPAGFFHPNVYPSGTVCLSILNEDSGWRPAITVKQILVGIQDLLDQPNPD
DPAQTDGYQLYMQDEFEYKKRVKQQAKQYPALL

>SlUBC19
MTSASASSRKVLSKIACNRLQKELMEWQVNPPAGFKHKVTDNLQRWIIEVNGAPGTLYANEMY
QLQVDFPEHYPMEAPQVVFIHPAPLHPHIYSNGHICLDILYDSWSPAMTVSSICISILSMLSSSTVK
QRPADNDRYVKNCKNGRSPKETRWWFHDDKV

>SlUBC20
MASKRILKELKDLQKDPPTSCSAGPVGEDMFHWQATIMGPSDSPYAGGVFLVTIHFPPDYPFKP
PKVAFRTKVFHPNINSNGSICLDILKEQWSPALTISKVLLSICSLLTDPNPDDPLVPEIAHMYKTDKS
KYEGTARSWTQKYAMG

>SlUBC21
MASASPSQASLLLQKQLKDLCKKPVDGFSAGLVDESNLFEWSVTIIGPPETLYEGGFFNAIMSFPQ
NYPNSPPTVKFTSEVWHPNVYSDGKVCISILHPPGDDPNGYELASERWSPVHTVESIMLSIISML
SSPNDESPANVEAAKEWRDNRDEFKKKVSRCVRRSQEMT

>SlUBC22
MASTSPSQASLLLQKQLKDLCKKPVDGFSAGLVDESNLFEWSVTIIGPPETLYDGGFFNAIMSFP
QNYPNSPPTVKFTSEIWHPNVYSDGKVCISILHPPGDDPNGYELASERWSPVHTVESIMLSIISM
LSSPNDESPANVEAAKEWRDNRDEFKKKVSRCVRRSQEMM

>SlUBC23
MINLIKVREKQKEAAENAGSKTPIKEQTASKLRVHRDISELTLPSICTIEFPNGKDDLMNFEVSIKPD
EGYYHDGEFPFKFEIPILYPHDAPKVKCKIKVYHPNIDYDGNVCLNILREDWKPVLNINAVIYGLIHL
FTEPNHEDPLNPEAADELRDNPRSFESHVRSAMWGESVHGLTYDQQNIDLEGNVCLNILREDW
KPILNINAVIYGLIHLFTEPNYEDPLNHEAADELRDNPKMFDSNVRRTMWGGHMHDVYFDRVL

>SlUBC24
MASKRILKELKDLQKDPPTSCSAGPVAEDMFHWQATIMGPTDSPYAGGVFLVSIHFPPDYPFKP
PKVAFRTKVFHPNINSNGSICLDILKEQWSPALTISKVLLSICSLLTDPNPDDPLVPEIAHMYKTDRA
KYETTARSWTQKYAMG

>SlUBC25
MAEKACVKRLQKEYRALCKEPVSHVVARPSPNDILEWHYVLEGSEGTPFAGGLYYGKIKFPPEYP
FKPPGISMVTPNGRFMTHKKICLSMSDFHPESWNPMWSVSSTGLLSFMMDTSPTTGSVTTTV
AEKQKLAKTSLAFNCKNPTFRKLFPEYVEKYEEQQLLVHPDQEQVSSMPTQAEISSPLLDGLNSV
EPHKDMENQRRKSLPTWLLLLLVSIFGVVMALPLLQL

>SlUBC26
MSTPARKRLMRDFKRLQQDPPAGISGAPQDNNIMLWNAVIFGPDDTPWDGGTFKLTLQFSED
YPNKPPTVRFVSRMFHPNIYADGSICLDILQNQWSPIYDVAAILTSIQSLLCDPNPNSPANSEAAR
MFSENKREYNRRVREIVEQSWTAD

>SlUBC27
MVDLARVQKELHECNRDVQVSGINVTLKGDSLTHLIGTIPGPVGTPYEGGTFKIDITLTDGYPFEP
PKMKFATKVWHPNISSQSGAICLDILKDQWSPALTLKTALLSIQALLSAPEPDDPQDAVVAQQYL
REHQTFVGTARYWTETFAKTSTLAADDKIQKLVEMGFPEAQVRSTLEANGWDENMALEKLLSS

>SlUBC28
MASKRILKELKDLQKDPPTSCSAGPVAEDMFHWQATIMGPTDSPYAGGVFLVSIHFPPDYPFKP
PKVAFRTKVFHPNINSNGSICLDILKEQWSPALTISKVLLSICSLLTDPNPDDPLVPEIAHMYKTDRA
KYETTARSWTQKYAMG

>SlUBC29
MDFIVLILYSILLNVLNSDMLYLTDMVQSDWEGGYYPMTIHFSENYPFMPPKCKFPQGFFHPNV
YPSGKVCLQSLFNQGHGWSPTITVKEILIAIPYLLDWPSPNYKAQTDSYNMYIQDNAEYRRRVRQ
QAEQYPAFF

>SlUBC30

MAQEARLNLRMQRELKLLLTDPPPGASFPSLTSSSSLSSIHALIGGPEGTVYAKGHFKLKIQIPERYP
FQPPIVTFLTPIYHPNIDNGGRICLDILNLPPKNRELGSHLTFQLSQVYACYVSLILMMGCMTRARS
TNTIDKHLTTRQDQLKSTPCLEHVILPVMIKKSELLQMQEKELLKSKNIYQNLRSQIILNRKGLCGLS
RKLSLDSAGRAKRHNGETASEVPIDHILNKQTEVSKQGMEEFPIECDLNQDEAQQRTKKLSSDIV
GTYKVRNGEKNSMAKTNCSASLEPQSI

>SlUBC31
MDVEIEEISAHDGSVKVKDNKEVMTEDNPDTVAGSTPGSTDSGIKNSSNLDITFHEDENDGDD
GLDDCDDMSNYDDDDDDYMYYDDEEDECDYLSMQAQFDNVDLPAGVEATVSWLNEPAPSSK
VSSQASSSSHLAGAQTLNSTLSEHASSSFAQVPASSSSLVSGGSNSCGKEEVTEDELMKKYRSFKH
FDVVEDFSDHHYSNLGVKGQQPPKAWSKKVQDEWKILENDLPDTIYVRVYEARMDLLRAVIIGP
QGTPYHDGLFVFDVLFPQNYPDVPPMVYYYSGGLRLNPNLYDCGKVCL

>SlUBC32
MANSNLPRRIIKETQRLLSEPAPGISASPSEDNMRYFNVMILGPTQSPYEGGVFKLELFLPEEYPM
AAPKVRFLTKIYHPNIDKLGRICLDILKDKWSPALQIRTVLLSIQALLSAPNPDDPLSENIAKHWKS
NEVEAVETAKEWTRLYASGA

>SlUBC33
MASKRIQKELKDLQKDPPASCSAGPVGQDMFHWQATIMGPSDSPFSGGVFLASIHFPPDYPFK
PPKVSFKTKVFHPNINSNGSICLDILKEQWSPALTVSKVLLSICSLLTDPNPDDPLVPEIAHMYKTD
RPKYESTARSWTQKYAMG

>SlUBC34
MASKRILKELKDLQKDPPTSCSAGPVAEDMFHWQATLMGPSDSPYAGGVFLVTIHFPPDYPFKP
PKVAFRTKVFHPNINSNGSICLDILKEQWSPALTISKVLLSICSLLTDPNPDDPLVPEIAHMYKTDKS
KYEATARSWTQKYAMG

>SlUBC35
MMSDYKVEMINDGMQEFFVEFHGPKESPYLGGVWKVRVELPDAYPYKSPSIGFVNKIYHPNVD
EMSGSVCLDVINQTWSPMFDLVNVFEVFLPQLLLYPNPSDPLNGEAAALMMRDRTTYDQKVK
EYCEKYAKKEDAGAPPEEKSSDEELSEEENVSSDDELAGKADP

>SlUBC36
MFHWQATIMGPPDSPYAGGLFLITIHFPPDYPFKPPKVAFRTKVFHPNINSNGSICLDILKEQWSP
ALTISKVLLSICSLLTDPNPDDPLVPEIAHMYKTDRNKYETTARSWTQKYAMG

>SlUBC37
MASSQAALLLQKQLKDLNRHPVDGFSAGLVDENNVFEWSISIIGPPETLFDGGFFNATMSFPTD
YPNNPPTVKFTTEIWHPNVYSDGRVCISILHPPGDDPNHYELASERWTPVHTVESIMLSIISMLTS
PNDESPANVDAAKEWRDNRAEFVKKVKRCVRKSQEML

>SlUBC38
METHKQVAAYVSENSKKRVFPGGSAIDVEVVEISRPTNWSSKSKTPKQHKKVSFHEIIDVDVEEN

LSDVKRSSGNVKFSGKGKDILVGNGSSGNSGPVDAVQSSKKNCFSSSIKKKNCLSASNSPIIIDEFG
SDVLFGADEHMDMYYDDLVYSDYAVLQAHFDNMDIPPGVEAPIPWMPGPMEEQMVSTTTST
SGRDVPGAVRNQSASFSSSTLVGQPTQFGSSWSSPGPALGKEGQLVIGNSNEKSSKGVSNSKKIH
SSGGTASFDWNNPYNMYEGSSSHYGKKLRSSGATTSYGSGNQTTPIGPYFPNSVGGPVSDMKN
LFLNPTTPIAAGITHASPAMMNYFPLSLHKGATTAGCPSIPSGPACNGEQHRNVDEILKNFQAFK
KFDTVEDHSDHFYSRQASSGNLPSKNWAKKIQEEWKILENDLPDTIFVRVYESRMDLLRAVIMG
ADGTPYHDGLYFFDVFFPSNYPNVPPLVHYHSFGLRINPNLYNCGKVCLSLLNTWTGQGKEKWI
PRASTMLQVLVSIQGLILNAKPYFNEPGYANTGGTARGDQSSLQYNENTYILNLKTMVFSMRRPP
KYFEDFVLGHFFRSAQDILVACKAYTDGAQVGSLVRGGVQDVDEGDKSCSPTFKASLAGFIKTVID
TFKEIGVKDCDKFLHLTQNGTE

>SlUBC39
MANSNLPRRIIKETQRLLSEPAPGISASPSEENMRYFNVMILGPTQSPYEGGVFKLELFLPEEYPM
AAPKVRFLTKIYHPNIDKLGRICLDILKDKWSPALQIRTVLLSIQALLSAPNPDDPLSENIAKHWKS
NEAEAVETAKEWTRLYASGA

>SlUBC40
MASKRILKELKDLQKDPPTSCSAGPVGEDMFHWQATIMGPPDSPYAGGVFLVTIHFPPDYPFKP
PKVAFRTKVFHPNINSNGSICLDILKEQWSPALTISKVLLSICSLLTDPNPDDPLVPEIAHMYKTDRA
KYESTARSWTQKYAMG

>SlUBC41
MSSPSKRREMDLMKLMMSDYKVEMINDGMQEFYVHFHGPAESPYHGGVWKIKVELPDAYPY
KSPSIGFINKMYHPNVDEISGSVCLDVINQTWSPMFDLTNVFEVFLPQLLLYPNPSDPLNGEAAA
LMMRDRTAYEQRVKEYCQKYAKPEDVGAVPEDKSSDEELSEAEYDSDDEAMAGPVDP

>SlUBC42
MQCCFFQFLLLVTLVFAGPYHGGVWKIKVEIPDAYLHKSTSIGFINKMYHPNVNEISGSVCLDVIN
HTWSPMFDLTNVFEVFLPQLLLYPNPSDPLNGEAAALMMRDRTSYEQRVQRYNDPYQLLIC

>SlUBC43
MQYCFFQFLLLVTLVFAGPYHGGVWKIKVEIPDAYLHKSTSIGFINKMYHPNVDEISRSVCLDVIN
QTWSPMFDLTNVFEVFLPQLLLYPNPSDPLNGEAAALMMRDRTSYEQRVQRYNDPYQLLICYK
NLCLL

>SlUBC44
MATNENLPPNVIKQLAKELKNLDDSPPEGIKVGVNDDDFSTIFADIEGPAGTPYENGVFRMKLILT
HDFPHSPPKGYFLTKIFHPNIASNGEICVNALKKDWSPSLGLRHVLMVVRCLLIEPFPESALNEQA
GKMLLDNYDEYARHARLYTSIHAKPKTKLKTGAISESTTALNVGQTHTSLCNVDQKTVVSGVAPL
QQPSPLSPTANIVKGGNNLDQPLTADTAVSGSAAPPSLTMKKETGLAKLPADKKKIDARKKSLKRL

>SlUBC45
MATMNSGNNSNTPATAAPVIPSPKQTQTTVKTVDTQSVLKRLQSELMALMMSGDSGISAFPEE
DNIFCWKGTITGSKDTVFEGTEYKLSLSFPADYPFKPPKVKFETGCFHPNVDVYGNICLDILQDKW

SSAYDVRTILISIQSLLGEPNISSPLNTQAAALWCNQEEYRKMVEKLYKPSV

>SlUBC46
MQASRARLFKEYKEVQREKTADPDIQLVCDDSNIFKWTALVKGPSETPYDGGVFQLAFSVPEQY
PLQPPQVRFLTKIFHPNVHFKTGEICLDILKNAWSPAWTLQSVCRAIIALMAHPEADSPLNCDSG
NLLRSGDIRGYQSMARMYTRLAAMPKKG

>SlUBC47
MSSPSKRRDMDVMKLMMSDYKVETINDGITEFNVEFHGPKESLYEGGVWKIRVELPDAYPYKS
PSIGFLNKIFHPNVDELSGSVCLDVINQSWSPMFDLLNVFEVFLPQLLLYPNPSDPLNGDAASLM
MKDKNQYEQKVKEYCERYAKKENVVGTPKDDSDDEISEEEFSGQSESDDEVVGHADP

>SlUBC48
MELPPSRYFSQNSKKRVFPGGSSTNVQVPEVSSTIARISKSENAKQKEPIHHGDVDMKGGDNDV
MFIDGNTGSRGKGKDFLELSLGYGDSAYSVGPSQVQSSKKHCPSESGEVPANLFYEDNPSIDMYF
SELANYDYAILQSHFDHMDTPPGVEVPIPWMSGFAKAKMASATTSTSSTSKALSSPFGRETPEVL
KYPSTSIYDLNHIFKPTPVELSSSSLGPAVVEGKFSAKANSKGICFKEQNGSTNVSPGVEKSLNVQG
SHLRRKIHLSPGTVSQSWHTPSIPGGVPFPTAHFVPPPMPSWMNLPPNMPTAQASSGFMLAP
GAMNPLPLHQVHPGFMLAPGDMNLLPLHQVHPGFMLATGDMISLPLEQVHPGYIIPPDTMN
YFLPELLSTGPVLAPGAMHYFHQEMDHELWTHGPHNSASLSLQCESAPSKEQHGNLGESLKNF
RLFKKFDTVQDHSGHYFSGLASHDNLASKSCAKRILEEWKILEKDLPDTIFVRVYETRMDLLRAVII
GADGTPYHDGLFFFDVYFPSNYPNVPPHVHYHSFGLRINPNLYECGKVCLSLLNTWDGRGKEK
WIPGESTMLQVLVSIQGLILNAKPYFNEPGYARMNGSASGERNSLRYNENTCIFNLKTMVYCMR
RPPQHFEDFVIGHYFQSCQDILVACKAYMEGARVGSLVRGCVLGDGGKGGDKGGSRSFKAMLA
GFIGVLVEAFKKIGCEDCDKFLPLAEKASTRATPVKID

>SlUBC49
MASKRILKELKDLQKDPPTSCSAGPVAEDMFHWQATIMGPADSPYSGGVFLVTIHFPPDYPFKP
PKVAFRTKVFHPNINSNGSICLDILKEQWSPALTISKVLLSICSLLTDPNPDDPLVPEIAHMYKTDRS
KYETTARSWTQKFAMG

>SlUBC50
MSDGIARGRLAEERKSWRKNHPHGFVARPETGADGSVNLMVWNCSIPGKAKTDWEGGFYPIT
MNFSEDFPSKPPKCKFPQGFFHPNVYPSGTICLSILNEDSGWRPAITVKQILVGIQDLLDQPNPSD
PAQTEGFQLYMQDGAEYKKRVRQQAKQYPALVH

>SlUBC51
MIKLFKVKEKQRELAENANGKPPIKKQSAGELRLHKDISELNLPETCSISFPNGKDDLMNFEVTIR
PDEGYYVGGTFVFSFSISSIYPHEAPKVKCKTKVYHPNIDLEGNVCLNILREDWKPVLNINTIIYGLY
HLFTEPNHEDPLNHEAAAVLSDNPSLFESNVRRAMSGGYVGETFFQRCI

>SlUBC52
MAEDKYNLKNPAVKRILQEVKEMQSNPSDDFMSLPLEENIFEWQFAIRGPRDSEFEGGIYHGRI
QLPAEYPFKPPSFMLLTPNGRFETQTKICLSISNHHPEHWQPSWSVRTALVALIAFMPTSPNGAL

GSLEYTKEERRVLAIKSRDVAPRYGTPERQKLIDEIHEYMLSKAPPVPQATTSPAPEEQNSNREEE
VQESPENPSNEATEERPPNPSVADTTTEERRELALDADPVQTPIEPPAVRPNEHRTLHTPQQSIPR
QADDRFFTWAAVGLTIAILALLIKKFMKANGHGAVFMDES
